# Graphical analysis for phenome-wide causal discovery in genotyped population-scale biobanks

**DOI:** 10.1038/s41467-020-20516-2

**Published:** 2021-01-13

**Authors:** David Amar, Nasa Sinnott-Armstrong, Euan A. Ashley, Manuel A. Rivas

**Affiliations:** 1 Center for Inherited Cardiovascular Disease, Stanford University, Stanford, CA USA; 2 Department of Biomedical Data Science, Stanford University, Stanford, CA USA; 3 Department of Genetics, School of Medicine, Stanford University, Stanford, CA USA

**Keywords:** Statistical methods, Genome-wide association studies, Population genetics, Mathematics and computing

## Abstract

Causal inference via Mendelian randomization requires making strong assumptions about horizontal pleiotropy, where genetic instruments are connected to the outcome not only through the exposure. Here, we present causal Graphical Analysis Using Genetics (cGAUGE), a pipeline that overcomes these limitations using instrument filters with provable properties. This is achievable by identifying conditional independencies while examining multiple traits. cGAUGE also uses ExSep (Exposure-based Separation), a novel test for the existence of causal pathways that does not require selecting instruments. In simulated data we illustrate how cGAUGE can reduce the empirical false discovery rate by up to 30%, while retaining the majority of true discoveries. On 96 complex traits from 337,198 subjects from the UK Biobank, our results cover expected causal links and many new ones that were previously suggested by correlation-based observational studies. Notably, we identify multiple risk factors for cardiovascular disease, including red blood cell distribution width.

## Abstract (web summary)

Mendelian randomization is a popular method to detect causal relationships between traits, but can be confounded by instances of horizontal pleiotropy. Here, the authors present a Mendelian randomization workflow which includes causal discovery analysis and filtering of genetic instruments based on their conditional independencies.

## Introduction

Causal inference from observational data is a fundamental objective that has been receiving increasing attention in multiple domains including biology, epidemiology, and economics. Graphical models are a cornerstone of causal inference as they explicitly describe the generating process of the observed data. These models contain functions that describe how values are assigned to each variable, possibly depending on the values of other observed or unobserved variables. These dependencies can be summarized in a directed graph, where an edge *X* → *Y* means that the function that determines the value of *Y* depends on *X*’s value. If the graph is acyclic, the joint distribution of the data can be represented as a Bayesian network (BN) that specifies the conditional probabilities of nodes given their parents^1^.

*Causal discovery* is a subfield of causal inference that focuses on finding evidence in data for the existence of a causal path between two or more variables^1,2^. This is an essential preliminary step as it can be used to justify the assumptions made by statistical analyses. Algorithms for causal discovery identify patterns of conditional independencies (CI) with theoretical justification for refuting candidate models that are unlikely to have generated the observed data. This process requires two assumptions (1) graphical d-separation and the *Causal Markov Condition* (CMC), and (2) *Causal Faithfulness Condition* (CFC)^2,3^ (see Supplementary Note 1 for formal definitions). CMC states that whenever a pair of variables *X* and *Y* are separated in the graph given a set *Z*, then *X* and *Y* are conditionally independent given *Z* in every compatible distribution. CMC has been proven to hold in acyclic models and in linear models with cycles (also called feedback loops)^4^. Some results support CMC in other cyclic cases^4,5^. CFC deals with the opposite direction: it assumes that a conditional independence (CI) in the observed distribution entails separation in the graph. CFC has theoretical justification in that the set of models that do not satisfy it are extremely unlikely (i.e., have a zero Lebesgue measure^2,6^).

In genetics, modern population-based cohorts often aggregate large datasets with extensive phenotypic and genotypic data of the same subjects. Due to their size and depth, these datasets offer new opportunities for discovery and inference of causal relationships between traits. Consequently, a plethora of methods have been suggested for causal inference using genetic data as instruments^7–12^. Most methods employ a graphical model called *Mendelian Randomization* (MR) in which for a given pair of phenotypes (*X*,*Y*) the effect sizes of the variants of *X* with both phenotypes are analyzed to estimate the causal effect of *X* on *Y*. When the effect sizes are estimated in the same dataset, we denote the analysis as a *single-sample* MR. When a different dataset is used to estimate the effect sizes of the exposures, we called it *two-sample* MR, which assumes that the two populations are compatible^13^. Standard MR methods assume linear effects and report a summary of a linear fit. IVW regression^14^, for example, uses inverse variance weights to average the causal estimates of the instruments^9^. MR assumes that the genetic variants are independent of confounders that affect both phenotypes, and that there is *no horizontal pleiotropy*: the instruments affect *Y* only through *X*^11,15^. These are strong assumptions that cannot be justified from the data when analyzing *X* and *Y* alone, especially if the genetic variant directly affects both phenotypes (which we call direct horizontal pleiotropy)^15^. This is exacerbated when analyzing multiple phenotypes because the assumptions are made for each *X*,*Y* pair. Implicitly, MR also assumes that the graph is acyclic. However, this problem can be mitigated in case-control situations, where the effects of the instruments on *X* are measured using the controls only^9^. Nevertheless, detecting cycles is particularly salient in the context of population biobanks, where medications, lifestyle changes, and variable temporal dynamics often confound the causal directions between measurements^16^.

Several methods have been proposed to address some of the limitations above. MR-Egger can model horizontal pleiotropy under the assumption that the effects of the instruments on the exposure and the outcome are independent^17^. MR-PRESSO accounts for horizontal pleiotropy by correcting for variants with outlier effects^8^. However, the assumption that outliers are not proper instruments and should be adjusted for may not hold in practice. Latent-causal variable analysis (LCV)^7^, assumes that there is a latent variable that mediates the genetic correlation between *X* and *Y* and then compares the genome wide effect sizes of association with both traits against each other to assess if one phenotype is fully or partially *genetically causal* for the other. LCV assumes acyclicity and does not estimate the causal effects. Regardless of recent progress, extant methods focus on inference for a single trait pair from the marginal summary statistics and are limited for causal discovery by their unidentifiable assumptions.

Other causal inference methods in genomics have been proposed to either mitigate the limitations of MR or to model larger graphical structures. For example, network MR analyzes an exposure and an outcome together with a mediator^18^. Multivariate MR can model multiple exposures jointly for the same outcome^19^. While these methods can provide accurate results, they require adding assumptions to the standard MR model. CAUSE is a recent extension of MR that uses genome-wide summary statistics to model causal effects while accounting for pleiotropy^20^. Another type of algorithms address gene network inference by joint analysis of genetic variants and gene expression data in order to learn a large-scale graphical model with causal links among genes^21–23^. Recently, Howey et al. explored similar methodology as an alternative for MR^24^. They showed that learning BNs among phenotypes while including genetic variants as anchors can improve upon MR. However, this method does not provide a clear way to select edges as it simply weighs all phenotype pairs by the proportion of times they were connected in bootstrap repeats.

In this work, we leverage the theoretical framework of causal discovery to enhance and streamline MR-based analysis. Our flow, Causal Graphical Analysis Using GEnetics (cGAUGE) first identifies unique CI patterns in the data and then uses them for: (1) filtering genetic instruments for downstream MR analysis, and (2) Exposure-based Separation (ExSep): an algorithm for causal discovery that does not require selecting genetic instruments in advance. The theoretical justification of these algorithms holds even in the presence of unobserved confounders and cycles. We present extensive simulations to illustrate how cGAUGE improves upon MR and BN methods by reducing their empirical false discovery rate by up to 30%. We apply cGAUGE to 67 complex traits (including 41 biomarkers) and 29 diseases using data from 337,198 subjects from the UK Biobank^25,26^. We find many expected causal links and new ones that were previously speculated in correlation-based observational studies. These new discoveries include causes of behavioral phenotypes and multiple risk factors for cardiovascular disease, including red blood cell distribution width (RBW), which is discovered using our ExSep (non-MR) test.

## Results

### cGAUGE: A novel pipeline for causal discovery using genetic instruments

**Fig. 1:**
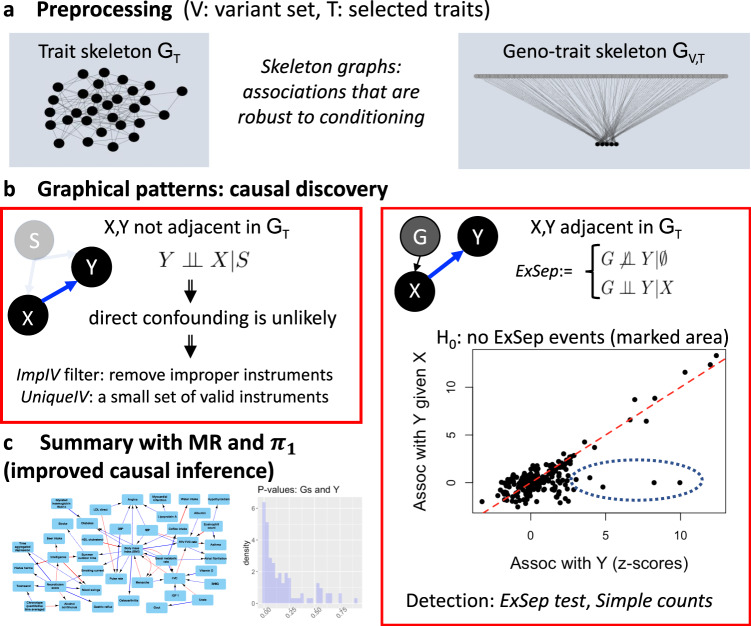
The cGAUGE workflow for causal discovery. We analyze genetic and phenotypic data collected from independent subjects. **a** We first preprocess the data to infer skeleton graphs: graphs that represent associations that are robust to conditioning. Based on causal inference theory^1,2^, surviving associations contain the subset of true causal links. We learn two skeletons: *G*_T_ among the phenotypes (ignoring the genetic data in the process), and *G*_V,T_ between the variants and the phenotypes. **b** We then analyze the edges and the non-edges of *G*_T_ separately. We present methods that use *G*_V,T_ to filter out improper instruments (ImpIV) or identify unique proper instruments (UniqueIV). While their theoretical justification is pertinent to *G*_T_ non-edges, we illustrate using simulations how they reduce the empirical FDR when applied to all phenotype pairs. For *G*_T_ edges we present an analysis based on *ExSep* events: associations between genetic variables and a trait *Y* that “disappear” once conditioned on a new phenotype *X* (i.e., *p* > *p*_2_). Under our *local faithfulness* assumption these patterns are evidence for a causal link from *X* to *Y*. The ExSep model selection test is a method to analyze all genetic variables under the null hypothesis of no ExSep events. **c** Finally, we utilize our results for improved inference using Mendelian Randomization (MR) and also utilize the *π*_1_ estimate for each exposure–outcome pair. This score quantifies the consistency of the associations between the exposure’s instruments and the outcome, which can be used to flag potential false positive causal links.

We present a new pipeline, cGAUGE: Causal Graphical Analysis Using GEnetics. cGAUGE is a set of tools that utilize CI tests for improving causal inference among traits using genetic variables (see Fig. 1 for an overview, and Supplementary Note 1 for a formal explanation). cGAUGE takes as input the individual level data of a population-based biobank, a CI test (e.g., using linear or logistic regression), and two *p*-value thresholds: *p*_1_ for rejecting the null of CI, and *p*_2_ for accepting it (*p*_2_ > *p*_1_, values in between are considered unreliable). While standard statistical tests are not designed to accept null hypotheses, this is a standard assumption made by causal discovery algorithms for detecting independence^1,2^. Alternatively, cGAUGE can take the summary statistics of all marginal and conditional tests. These are assumed to be adjusted for exogenous variables including sex, age, and genetic principal components (we used the top five by default).

cGAUGE has two types of output. First, it provides a set of filtered genetic variables that can be used as instruments for an MR analysis for a given pair of phenotypes. While the theoretical justification of these filters is pertinent to a subset of the trait pairs, we illustrate using simulations that they work well in practice for all pairs. Moreover, cGAUGE flags the trait pairs that do not have these guarantees to help users in interpreting the results. Second, it identifies phenotype pairs with evidence for causal interactions based on a new statistical test that does not require setting a significance cutoff for instruments. We now give an overview of the different steps.

We first seek associations in the data that are robust to conditioning. This is a standard preprocessing step as these associations contain the correct (direct, non-mediated) causal links as a subset^1,2^. The results are summarized in graphs called *skeletons*: (1) *G*_T_ for all phenotype pairs that are significantly associated with *p* < *p*_1_ even when conditioned on pairs of other phenotypes, and (2) *G*_V,T_ for genetic variables vs. phenotypes, created by excluding (*G*,*X*) associations for which there exists a phenotype *Y* such that conditioning upon it (in addition to the exogenous variables) results in *p* > *p*_2_.

*G*_T_ separates the phenotype pairs into* edges* and *non-edges*. For non-edges we present two practical algorithms to filter the instruments of an exposure *X* when analyzing an outcome *Y*. The first is based on the observation that given the phenotypes that separate *X* and *Y* while learning *G*_T_, their adjacent genetic variants in *G*_V,T_ are not valid instruments when analyzing *X* and *Y* (see Theorem 2.1 in Supplementary Note 1). We call this filter *ImpIV* as it removes improper instruments. However, note that (1) there is no guarantee that the remaining instruments are valid and (2) by definition, for *G*_T_ edges, *ImpIV* does not change the set of instruments.

The second filter is based on the observation that genetic variants that are linked only to *X* in *G*_V,T_ are valid instruments for analyzing *X* and *Y* (see Theorem 2.2 in Supplementary Note 1). We call this filter *UniqueIV*, as it finds unique skeleton-based instruments. As this set of instruments is identified for an exposure regardless of the outcome, this set can be used for all MR analyses (i.e., including for *G*_T_ edges). However, note that UniqueIV may remove most if not all of *X*’s instruments, potentially limiting the statistical power of the downstream MR analysis.

Given a set of instruments discovered by taking all GWAS results of *X*, or after applying one of our filters above, the causal discovery process now relies on an MR analysis. However, we also compute *π*_1_: the proportion of non-null *p*-values (i.e., when examining the *p*-values of the association of *X*’s instruments with *Y*) under the assumption that the *p*-values follow the two-groups mixture model^27^: *p*-values or their inverse normal *z*-score follow a mixture distribution of nulls and non-nulls (see Supplementary Note 1). *π*_1_ can be estimated using empirical Bayes approaches^28^ and it directly measures the association significance consistency while avoiding some parametric assumptions made by MR (e.g., that all instruments are pertinent to the same linear effect). Our simulations below illustrate how the *π*_1_ estimates can be used to filter out false positives.

For an edge (*X*,*Y*) in *G*_T_ we show that if there are cases of genetic variants that are marginally associated with *Y* but are independent of *Y* given *X*, then this serves as evidence for *X* being a cause of *Y*. We denote this pattern as *ExSep*. The proof holds under the faithfulness assumption. However, we show that it is still valid even under a weaker and more realistic assumption that we call *local faithfulness*: the assumption that an emerging CI reflects having at least one pathway that is blocked in the true causal diagram (Supplementary Note 1, Theorem 2.1). We examined two approaches to test for ExSep events for a given (*X*,*Y*) pair: (1) *Naive*: using a simple threshold for the number of events with CI(*G*,*Y*) with *p* < *p*_1_ and CI(*G*,*Y*,*X*) producing *p* > *p*_2_, and (2) *Model selection* (*MS*) *test*: an approach that tests the null hypothesis of no class of ExSep variants.

Denote the *z*-scores of the associations of all genetic variables with *Y* as **z**_**1**_ and all the *z*-scores with *Y* given *X* as **z**_**2**_. Note that these are the inverse normal scores of *p*-values and not effects sizes. Each *z*-score can represent a *null* case of no association or a *non-null* case of a true association. Thus, this fits the two-groups model discussed above, which we assume for simplicity is a mixture of two Gaussians^29,30^. We can therefore model the joint distribution of **z**_**1**_ and **z**_**2**_ as a mixture of four bivariate normal distributions corresponding to all four combinations of null and non-null cases. However, under the null hypothesis that there are no non-null **z**_**1**_ cases whose **z**_**2**_ statistic is null, we can model the data using a mixture of three Gaussians only. Assuming that the marginal two-groups models are known and fixed, the unknown parameters of the null and non-null models are the correlations between **z**_**1**_ and **z**_**2**_ within each bivariate normal distribution, and the prior probability of each cluster. We use a grid-search heuristic to fit these models and compute their likelihoods. These are then used to test the null hypothesis using a likelihood ratio test (see Supplementary Note 1).

### Simulations

**Fig. 2:**
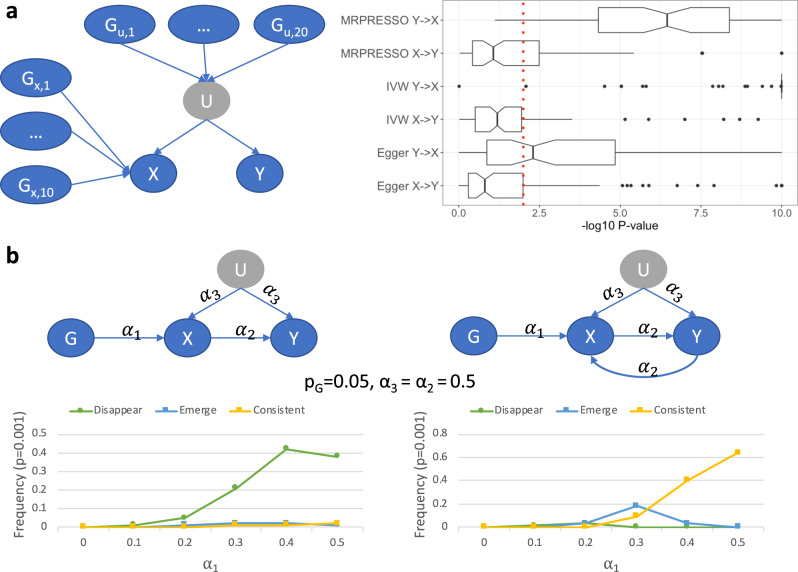
Simulations of simple graphs illustrate some limitations of MR and the faithfulness assumption. **a** When simulating from linear models from the presented graph (*n* = 2000 samples), MR methods tend to erroneously predict causal links between *X* and *Y*. The left panel shows the graph, where the gray node *U* is an unobserved common cause (confounder) of *X* and *Y*. The right panel shows the distribution of the (−log10) *p*-values of different MR methods when analyzing *X* and *Y*. Each boxplot shows the median, and first and third quartiles. The whiskers extend from the hinge to the largest and lowest values, but no further than 1.5 * (the inter-quantile range). As input for MR, summary statistics were computed using linear regression and instruments were selected using a *p* < 10^−04^ cutoff. **b** Simulations from simple MR models with a single binary instrument show violations of faithfulness in finite samples. Each line presents the frequency of different dependency patterns between the simulated genetic instrument *G* and the phenotypes *Y* and *X*. In all simulated cases *G* and *X* were significantly associated at *p* < 0.001, and are thus not shown. Disappearing associations: *G* and *Y* are associated marginally, but become independent when conditioned on *X* (*p* > 0.1). Emerging associations: *G* and *Y* are independent but become associated when conditioned on *X*. Consistent: *G* and *Y* are significantly associated with and without conditioning on *X*. Even though under faithfulness *G* and *Y* should be associated with and without conditioning on *X* (in both graphs, *X* acts as a collider on the path from *G* to *Y* through *U*), we see mixed results. Nevertheless, the detected associations occur only when a non-blocked path exists between *G* and *Y*, satisfying our refined local faithfulness assumption.

Consider the graph in Fig. 2a. There is no causal link between the two traits *X* and *Y*, but they are both affected by an unobserved confounder *U*. *X* has 10 binary instruments and *U* has 20. We tested the performance of MR-Egger, IVW, and MR-PRESSO on 100 datasets simulated from this graph (2000 samples each), with summary statistics computed using linear regression and instruments selected at *p* < 10^−04^. All causal quantities were sampled independently from the same uniform distribution $U_{\mathrm{{c}}} = U\left[ {\left( { - 0.9, - 0.1} \right) \cup \left( {0.1,0.9} \right)} \right]$, and each instrument was generated randomly with probability *p*_G_, sampled from *U*_g_ = *U*[0.05, 0.4] for each instrument. *U,X,Y* were all generated with standard normal errors. All three methods tended to erroneously predict a causal link from *Y* into *X* (e.g., at *p* < 0.01). Moreover, the tests for heterogeneity (IVW) or pleiotropy (MR-Egger, MR-PRESSO) produced insignificant results (*p* > 0.2 in >80% of the cases), illustrating that utilizing these additional tests could not salvage the analysis from making errors. In contrast, applying UniqueIV before MR results in no causal links between *X* and *Y* at the same 0.01 significance.

We also simulated data from a simpler MR model with a single genetic variant *G* with either an *X* → *Y* link or a feedback loop (see Fig. 2b). Over 100 repeats, we tested the association between *G* and *Y* with and without conditioning on *X*. The results had multiple cases in which the association between *G* and *Y* was not detected (at *p* < 0.001), illustrating how faithfulness may not hold in finite samples. This also demonstrates why local faithfulness is more realistic: it fits the graphs even if the CI test for *G* and *Y* given *X* is insignificant and *G* and *Y* are associated marginally.

We next simulated single-sample synthetic data of larger graphs over 15 continuous traits (with standard normal noise). Each trait had randomly selected ingoing and outgoing neighbors such that the expected in/out degree was set to ${\mathrm{{deg}}} \in \left( {1,1.25,1.5,1.75,2} \right)$. We then added *k* binary instruments for each trait, with *k* randomly selected from *U*[10, 20]. To add horizontal pleiotropy, for each instrument we decided whether it should have additional adjacent traits (in the true causal graph) at random with probability $p_{{\mathrm{{pleio}}}} \in \left( {0,0.1,0.2,0.3,0.4} \right)$, and if so, we added between 1 and 10 additional adjacent traits (selected randomly and uniformly). To summarize, as deg increases the generated graph has more cycles, and as *p*_pleio_ increases we are more likely to have violations of the MR assumptions. When generating datasets, causal quantities and binary instruments were generated using the *U*_c_ and *U*_g_ distributions above. For more details on how to simulate the data see Supplementary Note 1.

**Fig. 3:**
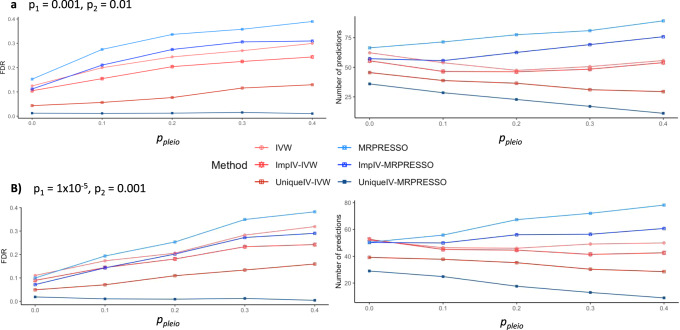
Mean number of discoveries and empirical false discovery rates (FDR) of Mendelian randomization methods in simulated data from graphs with 15 continuous traits. The underlying causal diagram was generated such that the expected in- and out-going degrees of the traits were 1.5. All simulated graphs contained cycles. For each trait we added between 10 and 20 binary instruments (uniformly, i.i.d). To add horizontal pleiotropy, for each instrument we decided whether it is horizontally pleiotropic or not with probability *p*_pleio_, and if so, we added between 1 and 10 links into additional traits (uniformly, iid). When generating datasets, the traits had standard normal noise, causal quantities were randomly and uniformly sampled such that their absolute value was between 0.1 and 0.9, and binary instruments were generated randomly with a probability between 0.05 and 0.4. The plots show the mean results of the simulations for different *p*_pleio_ values (e.g., the mean of the empirical FDR over the simulated graphs). Discoveries from each statistical test were done at a 10% significance level after adjusting for FDR using the BY algorithm. When two methods have a similar empirical FDR, greater number of predictions correspond to greater power. **a** Results with *p*_1_ = 0.001 and *p*_2_ = 0.01. **b** Results with *p*_1_ = 1 × 10^−05^ and *p*_2_ = 0.001. MR-Egger is not presented as it consistently had greater empirical FDR values than the other methods.

Figure 3 shows examples of the effects of our instrument filters from graphs generated with deg = 1.5. To test the effects of our filters even on skeleton edges, the MR methods were run on all trait pairs using a 10% BY FDR threshold^31^. The figures show the number of discoveries and their empirical FDR. MR-Egger is not presented as it consistently had greater empirical FDR values. The results of all MR methods and all possible combinations of deg, *p*_pleio_, *p*_1,_ *p*_2_ are available in Supplementary Data 1 and 2. All MR-methods, when run on the set of all exposure-associated instruments, had multiple cases of unreasonably high mean empirical FDRs, which was as high as 40%. Both ImpIV and UniqueIV consistently lowered the empirical FDR of IVW and MR-PRESSO. ImpIV’s effect was moderate in many cases, whereas UniqueIV had reasonable empirical FDR (<15%) whenever $p_1 \le 0.001$. Supplementary Fig. 1 shows the same simulations as in Fig. 3 but with a 1% FDR cutoff. The empirical FDR of the MR methods still remains unreasonably high with high horizontal pleiotropy (>25%). It also illustrates that using UniqueIV with MR-PRESSO is conservative as its empirical performance remains far below the FDR threshold used.

Moving to non-MR methods, we tested the performance of the ExSep-based approaches. Supplementary Fig. 2 shows an example (see Supplementary Data 1–4 for the full results). As in the MR analysis above, discoveries were made at 10% BY FDR adjustment. The results show that the naive analysis had poor performance when $p_{{\mathrm{{pleio}}}} {\,}> {\,} 0.1$. The MS test had a greater number of discoveries and a lower empirical FDR. As shown in Supplementary Data 3, there were a few cases in which the MS test had an FDR between 15% and 20%.

Finally, we examined the simple *π*_1_ estimates as a method for causal discovery. For a given threshold *t* we computed the number of discoveries and empirical FDRs for trait pairs with *π*_1_ > *t* but also the false positive rate among pairs with *π*_1_ < *t* (see Supplementary Data 5 and 6). The results show that trait pairs with *π*_1_ > 0.9 had low empirical FDRs (<10% in almost all cases). The few cases with FDR > 15% were all when *p*_2_ was set to too low values (*p*_2_ < 10^−4^). Trait pairs with *π*_1_ < 0.3 tended to be false positives (FPR > 90% in most cases). However, note that for *π*_1_ to be valid for discovery in practice, we must add an assumption that pleiotropic confounding occurs in low percentages when examining the variants associated with an exposure. Nevertheless, extremely low *π*_1_ scores can still be used for flagging potential false positives without additional assumptions.

We also compared cGAUGE to two recent methods: (1) CAUSE^20^, and the BN methods discussed in Howey et al. ^24^. We used our simulated data with *p*_pleio_ = 0 or *p*_pleio_ = 0.3, and deg = 1.5 (see “Methods” section for details). The comparison with CAUSE is presented in Supplementary Data 7. Overall, both CAUSE and cGAUGE improve upon other methods in terms of FDR control. However, we observed two advantages of cGAUGE: (1) it tends to have greater power, especially for low levels of horizontal pleiotropy, and (2) out of all compared methods only UniqueIV with MR-PRESSO keeps the empirical FDR lower than the predefined threshold in all cases. The latter is also correct in terms of worst-case performance (i.e., maximal empirical FDR observed over all simulated datasets), whereas CAUSE and UniqueIV with IVW can have 20% or greater.

When testing BNs with *p*_pleio_ = 0 the mean empirical FDR over simulated datasets was 8–8.5% among the top 10 predicted links, but was between 16% and 21.4% among the top 20 predicted links. With *p*_pleio_ = 0.3 the mean empirical FDR of either the top 10 or top 20 predicted causal links was >31%. These results again illustrate how extant methods are sensitive to high pleiotropy levels either from mediation (e.g., with *p*_pleio_ = 0) or horizontal pleiotropy (e.g., with *p*_pleio_ = 0.3).

### Results on the UK Biobank data

We applied cGAUGE to the 96 traits in Supplementary Data 8 using UniqueIV and MR-PRESSO as the base MR analysis at 10% BY FDR, and with a *π*_1_ > 0.25 cutoff. We tested *p*_1_ = 1 × 10^−6^, 1 × 10^−7^, or 1 × 10^−8^, and *p*_2_ = 0.01, or 0.001. These ranges are in line with MR publications for *p*_1_^32,33^, and settings of causal discovery algorithms for *p*_2_^34^. Comparing the choices for *p*_1_ and *p*_2_, the results are generally robust especially with *p*_2_ = 0.001 (>0.7 Jaccard coefficient, Supplementary Fig. 3). All trait pair results and the discovered instrument sets are available in Supplementary Data 8–16.

**Fig. 4:**
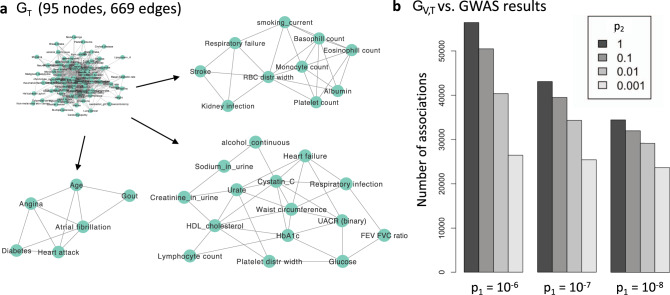
Inferred skeletons that represent associations that are robust to conditioning. **a** The skeleton inferred among the phenotypes (*G*_T_). The edges represent phenotype pairs that remain associated at *p*_1_ < 1 × 10^−7^ when conditioned on other phenotypes. In this computation for a pair of variables *X* and *Y*, we tested the association between *X* and *Y* conditioning on all other phenotypes or all other phenotype pairs. Arrows point out to clusters detected with MCODE. **b** The effect of conditional independence filtering on genome-wide association analysis (GWAS) results. Here we show the effect of removing variant–phenotype pairs (detected in a standard GWAS) for which there exists another phenotype whom conditioning upon renders the association insignificant (>*p*_2_).

Figure 4a shows *G*_T_ inferred with *p*_1_ = 1 × 10^−7^, resulting in 669 edges and 95 nodes. Clustering using the MCODE algorithm^35,36^ detected groups of densely connected related phenotypes. Changing *p*_1_ to 1 × 10^−6^ resulted in a similar network and clusters (Supplementary Fig. 4). Analyzing *G*_V,T_, we observed that up to 42.5% of the original GWAS results can be filtered, depending on *p*_1_ and *p*_2_ (Fig. 4b). These surviving variants (per GWAS) are more likely to contain true direct causal loci as compared to those excluded. In addition, when using *p*_2_ = 0.001 all *p*_1_ values result in a similar *G*_V,T_.

**Fig. 5:**
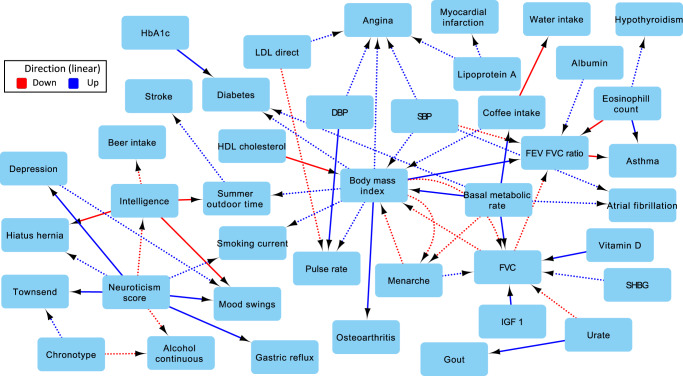
cGAUGE MR analysis with *p*_1_ = 1 × 10^−07^ and *p*_2_ = 0.001. All presented links were detected at 10% BY FDR correction and had *π*_1_ > 0.25. Solid arrows represent *G*_T_ edges (phenotypes whose correlation is robust to conditioning) and dashed arrows represent *G*_T_ non-edges. For simplicity, we excluded waist circumference and height, arrows out of diseases, and arrows into biomarkers. DBP diastolic blood pressure, SBP systolic blood pressure, HbA1c hemoglobin A1c levels, FEV forced expiratory volume, FVC forced vital capacity, IGF1 insulin like growth factor 1 levels, LDL direct low-density lipoprotein levels, HDL cholesterol high-density lipoprotein levels, SHBG sex hormone-binding globulin levels.

With *p*_1_ = 1 × 10^−7^, *p*_2_ = 0.001, we identified 290 causal links using our MR analysis (see Supplementary Data 9). Figure 5 shows a subset of the network that focuses on causal links from biomarkers into diseases and other phenotypes. Both LDL and lipoprotein A increase the risk for heart disease (e.g., angina *q* < 10^−17^, 0.58 log odds ratio (LOR)/mmol/L and 3.8 × 10^−3^ LOR/nmol/L, respectively)^37,38^. Other expected links include urate to gout (LOR/μmol/L 0.025, *q* = 1.97 × 10^−60^, MS test *p* = 9.64 × 10^−54^)^37^, body mass index (BMI) to pulse rate (*β* = 0.43, *q* = 1.85 × 10^−5^)^39^, BMI to diabetes (LOR/kg/m^2^ 0.25, *q* = 4.28 × 10^−15^)^40^, and basal metabolic rate (BMR) to diabetes (LOR/kJ, 4.8 × 10^−4^, *q* = 0.003)^41^. The network also contains causal links into behavioral phenotypes. For example, intelligence has a negative effect on mood swings (*β* = −0.15, *q* = 0.001*,* MS test *p* < 1 × 10^−100^), whereas depression has a positive effect (*β* = 0.5, *q* = 0.05).

While many links in Fig. 5 are expected, we detect multiple interactions that were previously suggested in observational studies. For example, we identify risk increasing factors for atrial fibrillation including blood pressure (SBP, LOR/mmHg 0.02, *q* = 0.098)^42^, and BMR (LOR/kJ 7 × 10^−4^, *q* = 5.9 × 10^−8^), which is in line with previous reports about correlations between the disease and metabolic syndrome^43^. Eosinophil count has risk increasing links to asthma (LOR/10^9^ cells/L 5.4 *q* = 4.4 × 10^−29^, MS test *p* = 3.46 × 10^−57^)^44,45^, and hypothyroidism (LOR/10^9^ cells/L 1.4, *q* = 0.1)^46^. The network also suggests that albumin increases FEV/FVC ratios (*β* = 0.002, *q* = 0.03). Serum albumin tends to be greater in normal individuals when compared to COPD patients or smokers, which tend to have lower FEV/FVC ratios^47,48^.

**Fig. 6:**
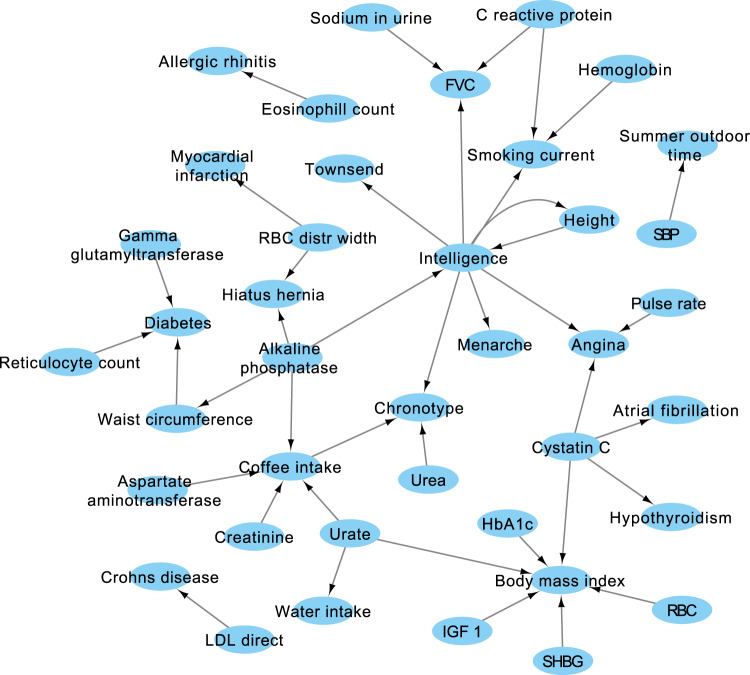
The top 40 links discovered uniquely by the ExSep MS test. The null hypothesis is that there are no *ExSep* events. That is, that there are no genetic variants that become independent of the outcome when adjusted for the exposure. An arrow represents an ordered pair with evidence for causal interaction with a significant MS test *p*-value even at 1% FDR adjustment. The MS-test does not report the direction of the causal effect, but can be significant even if the causal interaction is not linear. All links in the figure were not discovered by the MR analysis at 10% FDR adjustment, and are thus not in Fig. 5. SBP systolic blood pressure, HbA1c hemoglobin A1c levels, FVC forced vital capacity, IGF1 insulin like growth factor 1 levels, LDL direct low-density lipoprotein levels, RBC red blood count, SHBG sex hormone-binding globulin levels.

Figure 6 shows the top 40 pairs that had insignificant MR results and significant ExSep MS test results (all significant results are available in Supplementary Data 10). The network presents intelligence as a main hub that affects the Townsend deprivation score, age of menarche, smoking status, forced expiratory volume (FVC), angina, and height, which is also a feedback loop. Height-related causal links may result from temporal information, as discussed previously in O’Connor and Price^7^. For example, positive correlation between height and intelligence is a well-recognized phenomenon in children^49^, and nutritional status as a child is known to affect menarche age, height, lung capacity, and BMR^50–53^. The network also suggests RBW as a cause of myocardial infarction. The link between RBW and cardiovascular disease has observational evidence^54^.

## Discussion

In this work, we presented methods that utilize CI tests to enrich the causal analysis tool set of genetic biobanks. Our ImpIV and UniqueIV filters highlight which genetic instruments to use for MR analysis. They both start with the genetic variables detected in the GWAS. ImpIV filters out improper instruments but has no guarantees for the validity of the remaining set. UniqueIV removes more instruments and has guarantees about the remaining set, but it may be small and therefore less powerful in subsequent statistical analyses. Our theorems on which these filters rely mainly pertain to trait pairs that can be rendered independent by conditioning on other traits. However, given that they can still highlight valid instruments in all cases, they provide an alternative to the current practice in the community of using the GWAS results without filtering instruments while relying on assumptions alone.

We also provide a non-MR analysis for causal discovery based on the ExSep pattern: cases in which the association between genetic variables and the outcome are nullified when conditioned on the exposure. We provide a statistical test for the null hypothesis that no ExSep events exist when analyzing an exposure–outcome pair, which utilizes the set of all independent genetic variables and does not require selecting the instruments in advance. Our test is based on a grid search and future work can test if alternative optimization techniques improve the power of the test. In addition, unlike MR, this test does not infer the causal effect size. Future work can address integrating the results from our different analyses including the traits skeleton, the MR analysis, and the ExSep test. A major challenge here is to maintain the validity of our theorems while considering the uncertainty of skeleton edges under the same optimization framework.

Our instrument filters and the skeleton inference processes require *p*-value thresholds for either accepting or rejecting the null hypothesis of (conditional) independence. This is a general limitation of causal discovery algorithms as hypothesis testing was not developed for accepting null hypotheses^1,2,55,56^. Moreover, most causal discovery algorithms utilize a single *p*-value threshold and thus assume that for any statistical test they can decide between null and non-null. cGAUGE alleviates this issue in two ways. First, it leverages the large-scale nature of hypothesis testing with genetic variables using empirical Bayes techniques as these can sometimes justify inference about the proportion of null cases^27^. Second, we use two *p*-value thresholds such that CI events are used only if an association was first discovered with *p* < *p*_1_ and later had *p* > *p*_2_ when adjusting for additional variables.

In simulations we show that our methods have substantially lower empirical FDR. Specifically, our UniqueIV filter can reduce the empirical FDR by up to 30%, whereas our ExSep MS test has reasonable empirical FDR in almost all tested cases. In contrast, we observe that MR-Egger and BN learning have unreasonable empirical FDRs even with zero levels of added horizontal pleiotropy. IVW and MR-PRESSO can also have >20% empirical FDR (at 1% or 10% adjustment) as added horizontal pleiotropy levels increase (e.g., when 10% of the instruments are horizontally pleiotropic). We also illustrate that using the MR internal tests for pleiotropy cannot salvage the analysis when an unobserved confounder is well explained genetically (Fig. 2a). Our simulations were done using a single-sample, and when applied to the two-sample case, methods like MR-Egger and MR-PRESSO can have lower FDR. However, the observed empirical FDRs are still unacceptably high, and a two-sample MR can have additional challenges because of biases that lead to selection of improper instruments^13^. Finally, we observe that both cGAUGE and CAUSE reduce the empirical FDR as compared to all other methods. However, cGAUGE tends to have greater power, and it consistently keeps the empirical FDR in the desired level even in cases where CAUSE does not. These two methods, while being very different, represent a substantial progress in avoiding spurious results. Future studies can explore ways to integrate their ideas to further increase power.

We applied cGAUGE to a set of 96 phenotypes from the UKBB data. These were selected such that they cover many individuals and were not perfectly correlated as causal discovery may be invalid otherwise^2^. cGUAGE reports hundreds of causal links, most of which are expected. However, many of the identified links are novel and confirm previous suggestions from epidemiological observational studies that reported correlations with no causal inference. Notable examples include links from blood pressure to atrial fibrillation, serum albumin to lung function, and RBW to cardiovascular disease. These are only a few examples and we provide the results for all pairs in the supplementary material. There are two important considerations when interpreting large-scale causal networks. First, while we adjusted for population structure using the top genetic principal components, and included the Townsend deprivation score to account for socio-economic status, there may still be some errors in the output networks due to statistical errors. For example, even in our simulations of data with limited pleiotropy levels, the empirical FDR was not zero. Second, some significant MR results were filtered out using our *π*_1_ > 0.25 cutoff, including known false positives (e.g., HDL → angina^57^). *π*_1_ quantifies the proportion of non-null exposure instruments that are associated with the outcome. Both in theory and in simulations, low *π*_1_ suggests that the detected links may be false positives. Thus, it allows flagging problematic results.

Utilizing CI patterns is a unique property of our flow that is not covered by extant approaches that use genetic data. These tests require using the individual-level data and are thus not as easily available as the GWAS summary statistics that standard MR uses. However, if the summary statistics of these tests are provided, cGAUGE can be run without the individual level data. In our case, this amounts to all CI tests for roughly 50,000 genetic variants, which is a reasonable size dataset that can be shared by the community. Moreover, while our MR analyses in this paper are all based on single-sample MR, our UniqueIV and ImpIV filters provide static instrument sets that can be used and explored in future studies. Specifically, these can be used for two-sample MR, which requires learning the instrument set on one sample and estimating the causal effects on another. This analysis can help in reducing bias of estimated causal effects^58^. The same methodology can be used to improve multivariate MR as it also requires precise instrument sets (e.g., by requiring that the instruments are not directly linked to confounders)^19^. In addition, our instrument sets can be used as weights when interpreting the GWAS results of an exposure. UniqueIV variants are more likely to be causal than ImpIV-only variants. Variants that are removed by ImpIV can be down weighted as cGAUGE identifies evidence for a path to some outcome that is not through the exposure.

## Methods

### UK Biobank data

We used 805,462 directly genotyped variants from 337,198 white British subjects from the UK Biobank^25,26^. The MHC region was excluded (chromosome 6, positions 23–35M). Data were preprocessed as explained in ref. ^59^ with a small change: we excluded variants with a MAF < 1%. 96 phenotypes (traits and diseases) were selected for the analysis (see Supplementary Table 8). These were selected to cover the phenotypes analyzed by O'Connor and Price^7^, but additional traits that had large sample sizes were added.

### Biomarker data

Biomarker measurements from UK Biobank participants were adjusted for 83 covariates, including age, sex, their interaction, assessment centers, and technical factors^60^, except that Townsend Deprivation Index and principal components of the genotyping matrix were not included in the regression. Residuals from these regressions were used for downstream analysis.

### Single GWAS

Genome wide association analysis per phenotype was performed using PLINK (version 2.0a2)^61^. The baseline results for each each GWAS were adjusted for sex, age, and the top five genetic PCs. We also clumped the results using PLINK’s greedy approach with the following parameters: –clump-p1 0.0001, –clump-r2 0.1, and –clump-kb 500.

### Graph visualization

All networks were plotted using Cytoscape version 3.7.2^62,63^.

### Computing *π*_1_

When analyzing an exposure–outcome pair with a given set of exposure instruments, we examined the distribution of the *p*-values of the associations of the variants with the outcome. We computed the proportion of non-null *p*-values as a measure of association significance consistency. This measure is commonly used by FDR methods and it is estimated by comparing the observed distribution of *p*-values to a random uniform distribution. Specifically, we use the local FDR method implemented in limma (version 3.42.2)^64,65^.

### BN inference

We used the bnlearn R package (version 4.5) for inferring BN^66^. We implemented the analysis presented in Howey et al. ^24^: BN were inferred using the hill climbing algorithm using the Bayesian Information Criterion (BIC) as the objective function with 50 random restarts to address convergence into local optimum. Directed edges from the phenotypes into the genetic variants were not allowed (i.e., using the bl option of the algorithm). We used the BIC-CG discrete–continuous hybrid objective function that models continuous variables using a mixture of Gaussians. Discretization of the data instead of this option resulted in worse empirical FDR scores and was therefore excluded. Networks were averaged over 100 bootstrap repeats, where in each repeat the top inferred network was kept. For each phenotype pair, we computed the proportion of times they were linked in the networks and took either the top 10 or top 20 as the predictions of the algorithm.

### Other analyses

We tested MR methods by taking the GWAS summary statistics of each phenotype (without the MHC region) and ignoring the CI tests. For MR, given a *p*-value threshold (*p* = 1 × 10^−6^, 1 × 10^−7^, or 1 × 10^−8^) we select the top GWAS regions with MAF > 1%, clump them using PLINK, and use the filtered results as the instruments. We used the MendelianRandomization R package (version 0.4.2)^67^ to run MR-Egger and IVW. We used the MR-PRESSO implementation from the original publication^8^ (version 1.0) with outlier correction, and also report the results of its global test for pleiotropy.

### Robustness analysis

To measure the robustness of our pipeline we tested different values for *p*_1_ (1 × 10^−6^, 1 × 10^−7^, 1 × 10^−8^) and *p*_2_ (0.1, 0.01,0.001) and computed the Jaccard score for each network type between the different combinations of *p*_1_ and *p*_2_ (see Supplementary Fig. 3).

### Reporting summary

Further information on research design is available in the Nature Research Reporting Summary linked to this article.

## Supplementary information

Supplementary Information

Reporting Summary

Description of Additional Supplementary Files

Supplementary Data 1–10

Supplementary Data 11–16

## Data Availability

Code to simulate data and the UK-Biobank summary statistics used to generate the results are available at https://github.com/david-dd-amar/cGAUGE/^68^. UK-Biobank data was retrieved using application 24983. The results from all analyses, including the instrument sets and pairwise Mendelian Randomization results are available in the Supplementary Data.

## Code availability

R implementation of cGAUGE is available at https://github.com/david-dd-amar/cGAUGE/. Code for preprocessing UK-Biobank biomarker data is available at https://github.com/rivas-lab/public-resources/tree/master/uk_biobank/laboratory-tests.
