# Supplementary material for: Graphical analysis for phenome-wide causal discovery in genotyped population-scale biobanks
Source: Nat Commun. 2021 Jan 13;12:350. doi: 10.1038/s41467-020-20516-2 (PMC7806647; doi:10.1038/s41467-020-20516-2)
Supplement: Supplementary file 1 — Supplementary Information [file 41467_2020_20516_MOESM1_ESM.pdf]

Supplementary Information: Graphical analysis for phenome-wide causal discovery in genotyped population-scale biobanks

Supplementary Figures

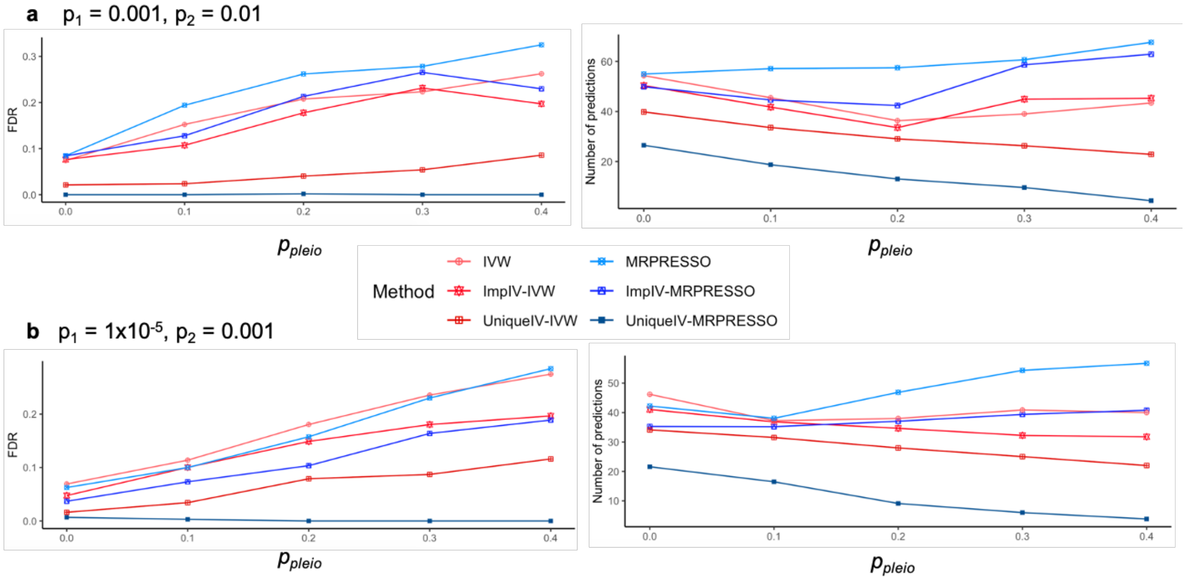

Supplementary Figure 1. Mean number of discoveries and empirical false discovery rates (FDR) of Mendelian randomization methods in simulated data from graphs with 15 continuous traits. The underlying causal diagram was generated such that the expected in- and out-going degrees of the traits were 1.5. All simulated graphs contained cycles. For each trait we added between 10 and 20 binary instruments (uniformly, iid). To add horizontal pleiotropy, for each instrument we decided whether it is horizontally pleiotropic or not with probability p_{pleio}, and if so, we added between 1 and 10 links into additional traits (uniformly, iid). When generating datasets, the traits had standard normal noise, causal quantities were randomly and uniformly sampled such that their absolute value was between 0.1 and 0.9, and binary instruments were generated randomly with a probability between 0.05 and 0.4. The plots show the mean results of the simulations for different p_{pleio} values (e.g., the mean of the empirical FDR over the simulated graphs). Discoveries from each statistical test were done at a 0.01 significance level after adjusting for FDR using the BY algorithm. When two methods have a similar empirical FDR, greater number of predictions correspond to greater power. **a** Results with $p_1 = 0.001$ and $p_2 = 0.01$. **b** Results with $p_1 = 1x10^{-05}$ and $p_2 = 0.001$.

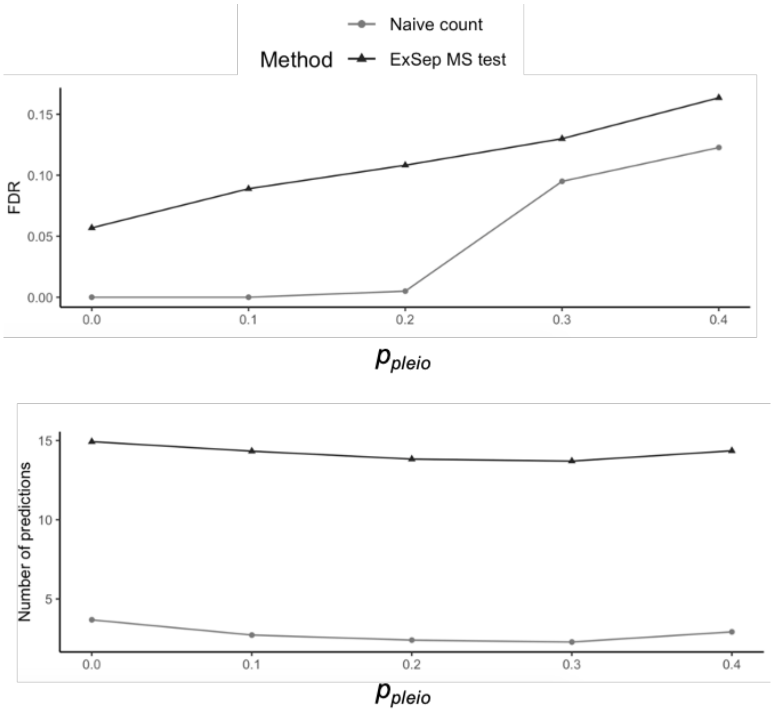

Supplementary Figure 2. Mean number of discoveries and empirical false discovery rates (FDR) of ExSep-based methods in simulated data from graphs with 15 continuous traits. The underlying causal diagram was generated such that the expected in- and out-going degrees of the traits were 1.5. All simulated graphs contained cycles. For each trait we added between 10 and 20 binary instruments (uniformly, iid). To add horizontal pleiotropy, for each instrument we decided whether it is horizontally pleiotropic or not with probability p_{pleio}, and if so, we added between 1 and 10 links into additional traits (uniformly, iid). When generating datasets, the traits had standard normal noise, causal quantities were randomly and uniformly sampled such that their absolute value was between 0.1 and 0.9, and binary instruments were generated randomly with a probability between 0.05 and 0.4. The plots show the mean results of the simulations for different p_{pleio} values (e.g., the mean of the empirical FDR over the simulated graphs). Discoveries from each statistical test were done at a 0.1 significance level after adjusting for FDR using the BY algorithm. When two methods have a similar empirical FDR, greater number of predictions correspond to greater power. Naive counts results were obtained with $p_1 = 1x10^{-05}$ and $p_2 = 0.001$. Note that the results here are not presented together with the MR methods (i.e., Figure 3 in the main text) because both the y-axes scale is different, and unlike the MR methods the ExSep test was applied only to skeleton edges and thus the total number of discoveries are not comparable between the two analyses.

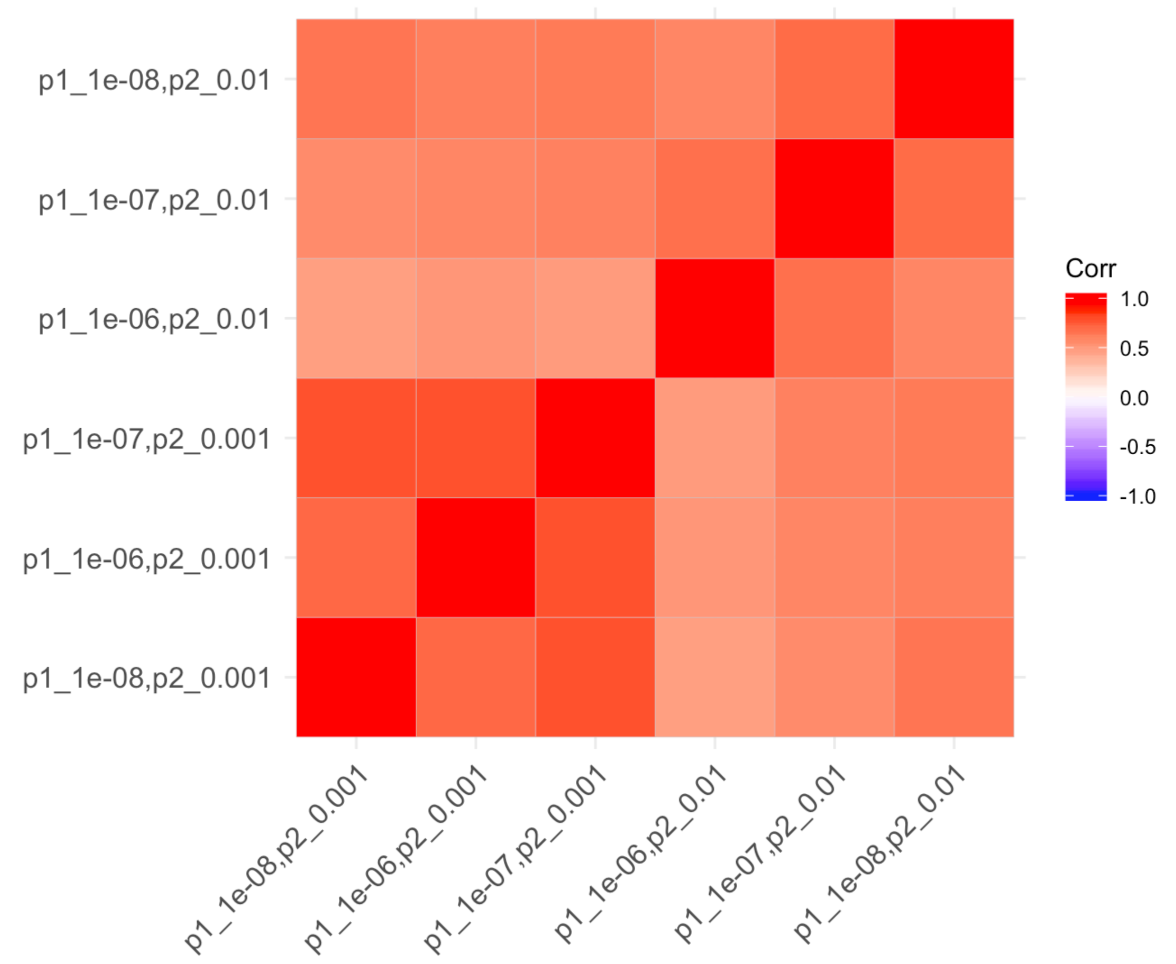

Supplementary Figure 3. Overlap matrix of the MR cGAUGE results with different p1 and p2 parameters. Each entry shows the Jaccard coefficient between the set of pairs inferred (size of intersection / size of union) at 0.1 FDR with MR-PRESSO as the base MR method, and UniqueIV as the instrument filter.

$\mathcal{G}_T$ (1e-06) 95 nodes, 719 edges

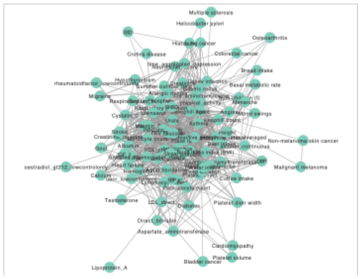

$\mathcal{G}_T$ (1e-06) Detected clusters:

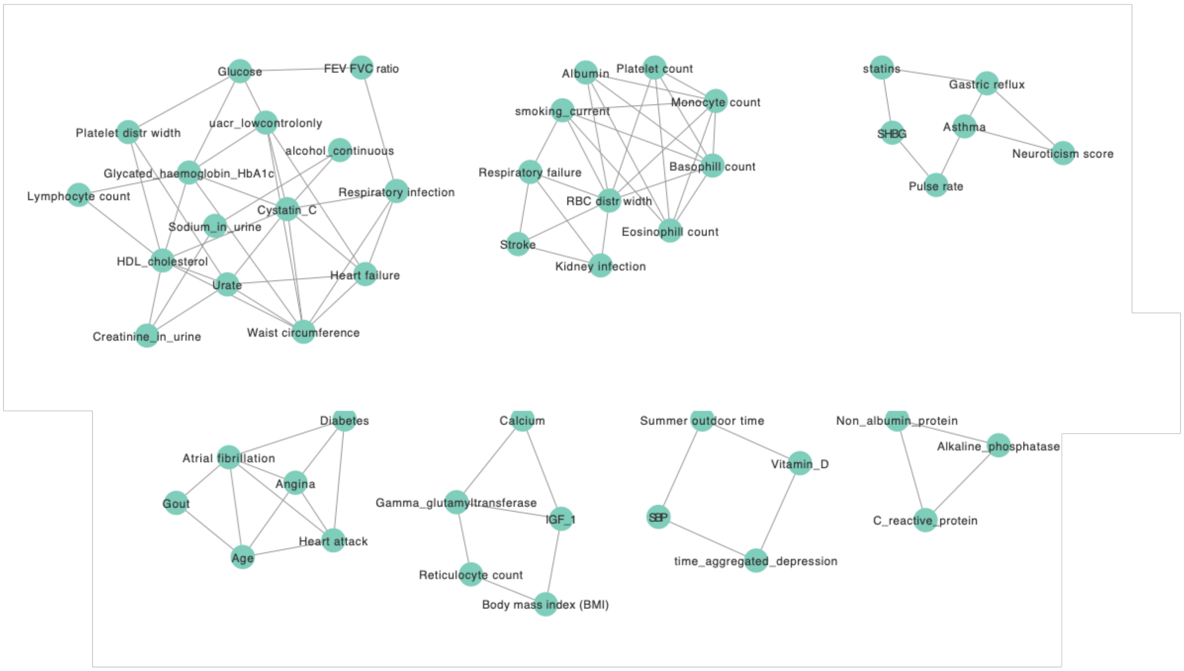

Supplementary Figure 4. Inferred G_T with $p_1 = 1X10^{-06}$. The edges represent phenotype pairs that remain associated at $p < 1X10^{-06}$ when conditioned on other phenotypes. Clusters detected by MCODE are presented below the main network.

Supplementary Note 1: A formal explanation of cGAUGE

1 Notation and background

We start with an overview of the theoretical results that provide the foundation for our algorithms. For a more thorough background on theory of causal inference see [1, 2].

General notation. We generally use the following notations unless stated otherwise. Graphs: calligraphic uppercase (e.g., $\mathcal{G}$). Distributions (including empirical): blackboard bold typeface (e.g., $\mathbb{P}$). Italic uppercase: a random variable. Italic bold uppercase: a set. Lowercase letter: a scalar or a realization of a random variable.

Graphs. A graph $\mathcal{G}$ is an ordered pair $\mathcal{G} =< \boldsymbol{V}, \boldsymbol{E} >$, where $\boldsymbol{V}$ is a set of nodes (or vertices) and $\boldsymbol{E}$ is a set of pairs of nodes. When two nodes are connected by an edge we say that they are adjacent. In an undirected graph, $\boldsymbol{E}$ is a set of unordered pairs, whereas in a directed graph $\boldsymbol{E}$ is a set of ordered pairs and an edge $< X, Y >$ can also be marked as $X \rightarrow Y$. If $\mathcal{G}$ is directed and $< X, Y > \in \boldsymbol{E}$ then we say that X is a *parent* of Y. We denote the set of parents of a node Y as $Pa(Y)$. In this paper we consider directed graphs without self loops, that is $\forall X \in \boldsymbol{V}$, $X \notin Pa(X)$. Generalizing parent-child relationships, we can naturally define descendants and ancestors.

An *undirected* path π between X and Y is a set of consecutive edges (independent of their orientation) connecting the variables such that no vertex is visited more than once. A *directed* path from X to Y is a set of consecutive directed edges from X to Y in the direction of the edges. A (directed) cycle in the graph occurs when there is a (directed) path from X to Y, and Y and X are also adjacent (i.e., $Y \rightarrow X$ in the directed case). A non-endpoint variable X in a path π is called a *collider* if and only if the edges around it have their arrowheads into X (i.e., $Z \rightarrow X \leftarrow Y$).

Causal models. We use the structural equation model with independent errors (SEM-IE) to describe a *causal model* [1]. This fits the standard assumptions made by current methods for genetic data analysis. Briefly, a causal model M is a pair $< \mathcal{D}, \Theta_{\mathcal{D}} >$ with a directed graph $\mathcal{D}$ over a set of variables $\boldsymbol{V}$. $\Theta_{\mathcal{D}}$ is a *parameterization* that assigns a function $x_i = f_i(Pa(X_i), \epsilon_i)$ for each $X_i \in \boldsymbol{V}$, where ϵ_i is an error term (also called disturbance) distributed independently of other error terms according to a distribution $P(\epsilon_i)$. Given all variables and distributions in a causal model, the observed data follow a joint distribution $\mathbb{P}$.

DAG factorization. The causal diagram $\mathcal{D}$ is the underlying graph that represents the direct causal interactions between the variables represented by the vertices $\boldsymbol{V}$ (some may be unobserved). If $\mathcal{D}$ is directed and acyclic (DAG) we say that a distribution $\mathbb{P}$ satisfies that *Markov*

property if it factorizes over $\mathcal{D}$ such that $f(\boldsymbol{X}) = \prod_{i=1}^{|X|} f_i(X_i|Pa(X_i))$, where f marks density functions. A similar definition can be used for discrete variables using probability functions instead of densities.

D-separation. Early work had established that some conditional independencies can be inferred from the graph structure alone regardless of the parameterization of the entire causal model [1]. When found, such discoveries constraint the set of possible distributions that are compatible with the underlying causal structure. The graphical rule is called *d-separation* and it is defined as follows. A set of variables $\boldsymbol{S}$ (possibly an empty set) d-separates a set of variables $\boldsymbol{X}$ from another set $\boldsymbol{Y}$, where $\boldsymbol{X}, \boldsymbol{Y}$, and $\boldsymbol{S}$ are all disjoint, if and only if every path from a node in $\boldsymbol{X}$ to a node in $\boldsymbol{Y}$ is blocked by $\boldsymbol{S}$. A path π between two nodes X and Y is blocked by a set of nodes $\boldsymbol{S}$, $X, Y \notin \boldsymbol{S}$ if and only if at least one of the following is satisfied: (1) $\boldsymbol{S}$ contains a node m that emits an edge in π (i.e., there is an edge $m \rightarrow m'$ in π), (2) there is a collider X' in π such that not X' nor any of its descendants are in $\boldsymbol{S}$.

We use $(X \perp\!\!\!\perp Y|\boldsymbol{S})_{\mathcal{D}}$ to mark the event that $\boldsymbol{S}$ d-separates two nodes X and Y in the graph $\mathcal{D}$. We use $(X \perp\!\!\!\perp Y|\boldsymbol{S})_{\mathbb{P}}$ to denote the case in which two random variables X and Y are conditionally independent given a set of variables $\boldsymbol{S}$ according to their joint distribution $\mathbb{P}$. The d-separation property of DAGs can be succinctly written: if $(X \perp\!\!\!\perp Y|\boldsymbol{S})_{\mathcal{D}}$ then $(X \perp\!\!\!\perp Y|\boldsymbol{S})_{\mathbb{P}}$ in any distribution $\mathbb{P}$ that is compatible (i.e., satisfies the Markov property) with $\mathcal{D}$.

D-separation in graphs with cycles. Although originally proven for causal models with DAG diagrams, the d-separation criterion was later proven for diagrams with cycles in two cases: (1) discrete finite variables in positive distributions [3], and (2) linear SEM-IEs [4]. Of note, the Markov property does not necessarily hold when cycles are introduced. Therefore, additional assumptions must be used. For example, in both cases above, the proofs require the assumption that the observed distribution is an *equilibrium.* Roughly, an equilibrium in this context is reached from an SEM when we start with a random sample of the errors and then simulate the SEM repeatedly until we achieve convergence. The set of values achievable in this process is called the *equilibrium distribution.* Fisher's fixed point method can be used to simulate the equilibrium distribution from an SEM-IE, but there is a closed form solution for simulating from a linear SEM-IE. In the discrete case, stronger assumptions are required as the process depends on the order of the equations [5]. Nevertheless, once the equilibrium is well-defined, d-separation holds [5, 6].

Causal discovery. The tight connection between causal diagrams and conditional independencies has been used to propose causal discovery algorithms. For DAGs, we used the *Markov condition* mentioned above, which also means that each variable X is independent of its non-descendants given its parents. For diagrams with cycles, while this property does not hold, the $d-separation$ rule is still used. However, two additional fundamental assumptions that seem plausible in practice and can be treated as axioms are used as well [2]: *minimality*, and

faithfulness. Minimality is the causal inference analog to Occam's Razor: we prefer simpler models when we consider alternatives that can explain the data. More formally, a causal diagram $\mathcal{D}$ satisfies the minimality condition with respect to a distribution $\mathbb{P}$ if the Markov property does not hold for every $< \mathcal{H}, \mathbb{P} >$, where $\mathcal{H}$ is a proper sub-graph of $\mathcal{D}$. Finally, faithfulness, (also called stability) assumes that all independencies embedded in the observed distribution $\mathbb{P}$ are stable and are invariant to changes in parameterization. Thus, it implies (together with d-separation) that $(X \perp\!\!\!\perp Y|Z)_{\mathbb{P}} \iff (X \perp\!\!\!\perp Y|Z)_{\mathcal{D}}$.

The assumptions above are especially important if we are to propose causal discovery approaches in the present of confounding by latent variables. We generally say that the effect of X on Y is *confounded* if there exists a third variable U such that $X \leftarrow U \rightarrow Y$. If U is unobserved the standard notation is $X \leftrightarrow Y$. We use $X \overset{\leftrightarrow}{\leftrightarrow} Y$ instead to avoid confusion with having a cycle $X \rightarrow Y$ and $X \leftarrow Y$. Presence of unobserved confounding adds complexity to the inference problem and limits our discovery power. Nevertheless, conditional independencies inferred from observed distribution are still powerful enough to constraint the set of plausible causal diagrams. When enough constraints are accumulated we can partially or completely infer causal diagrams.

It is important to note the fundamental difference between this algorithmic approach and standard statistical inference algorithms that optimize a global objective function such as training a Bayesian network using maximum a posteriori (MAP) or maximum likelihood (MLE) objectives. Here, the basic assumptions and the theoretical results are used as constraints in order to narrow down the set of possible causal diagrams. Thus, these algorithms rely heavily on the ability to statistically detect conditional independencies, and their output may be an only partially oriented diagram. Notable algorithms to perform such analyses are PC [4], IC* [1], FCI [2, 7], and CCD [8, 2]. Another notable example is the SAT-based optimization algorithm of [9] that extended the basic ideas to address the inference task as an optimization problem.

A few notable drawbacks of the algorithms above should be highlighted, especially for the case of analysis of large biobanks. First, these algorithms require exponential running times as results from all conditional independencies may be required. Second, most of these algorithms assume that the input contains a statistical oracle for testing conditional independence. Alternatively, this assumes that in practice there are no statistical errors, although some recent approaches had attempted to mitigate this issue by addressing the FDR of inferred edges [10, 11]. Nevertheless, the sequential nature by which these algorithms update the inferred structure implies that errors are propagated and accumulated. These two issues are crucial when biobanks are considered as we may need to analyze data from thousands of weak genetic variants and dozens of phenotypes. Our algorithms below build upon the main principles of these methods, integrating them with prior biological information about the nature of genetic variables.

Instrumental variable analysis. A special set of causal inference algorithms can be defined once *instrumental variables* are present. A variable Z is *instrumental* relative to (X, Y) if it is not independent of X and is independent of all variables that have influence on Y that is not mediated by X [1]. Using instrumental variables, we can use path-analysis methods to quantify causal effects associated with the edges of an SEM [12, 13]. In the genetic and epidemiological literature such analyses are often called *Mendelian Randomization* (MR), and the recent availability of genetic biobanks has led to increased interest in new MR methods. These methods are appealing for their ability to handle many instruments, reverse causality in some situations, and confounding effects [14]. Another common approach is to compute the overall genetic correlation (GC) between phenotypes. The recently proposed LCV method [15], uses higher moments of inferred summary statistics to move beyond standard GC to causal inference under a specific parametric model.

Using genetic instruments for causal inference process is promising as they act as markers that carry causal information flow. This is a unique property not generally achievable in causal inference from observational data, which explains the high popularity of MR and GC methods. However, extant methods are limited in that: (1) they assume that the instruments are known and are not confounded themselves (i.e., by population bias), (2) they are typically based on a parametric linear model, (3) they are sensitive to horizontal *pleiotropy*: confounding effects caused by the genetic instruments, and (4) these methods are limited to analysis of GWAS summary statistics and are therefore limited in the ability to utilize conditional independencies. In addition, for causal discovery these methods explore only a single type of graphical pattern. In our analysis we make use of constraint-based methodology to alleviate these issues and guide the causal inference process in large genetic biobanks.

2 Graphical analysis for causal discovery in genetic biobanks

2.1 Input data

We formalize our input as follows. We have two sets of features $\boldsymbol{V}$ (instruments), and $\boldsymbol{T}$ (phenotypes/traits/diseases) measured in n independent subjects. We denote the empirical distribution of the data as $\hat{\mathbb{P}}$. $\boldsymbol{V}$ is a set of genetic variants (typically SNPs) that are used as instruments for the causal inference process. $\boldsymbol{T}$ is a set of phenotypes, that are typically complex traits and diseases. Generally, $|\boldsymbol{V}|$ is large initially (>500,000), but can be substantially reduced once relevant variants are detected and LD-clumped (or pruned) per phenotype. $|\boldsymbol{T}|$ is much smaller. In our case we used the UK Biobank to obtain data of 337,198 white British subjects. We selected 96 phenotypes by taking those covered in [15] and some additional ones that had large sample sizes, see **Supplementary Table 1**. In addition, we assume that sex, age, and genetic principal components are exogenous variables. We denote their collective set

as $\boldsymbol{W}$, and assume that $\boldsymbol{W} \bigcap \boldsymbol{T} = \emptyset$.

Our analyses depend on three additional parameters: a test for conditional independence and the thresholds to make a decision about the results of such tests. Let $ci(X, Y, \boldsymbol{S})$ be a statistical test for conditional independence (i.e., a function that returns a p-value). The *null hypothesis* of this test is that $(X \perp\!\!\!\perp Y|\boldsymbol{S})_{\hat{\mathbb{P}}}$, and low p-values serve as evidence for the alternative $(X \not\perp\!\!\!\perp Y|\boldsymbol{S})_{\hat{\mathbb{P}}}$. By default, we use linear regression or logistic regression (for binary variables) for conditional independence tests. The two additional parameters are p_1, and p_2. When $ci(X, Y, \boldsymbol{S}) \leq p_1$ we conclude that $(X \not\perp\!\!\!\perp Y|\boldsymbol{S})_{\hat{\mathbb{P}}}$. When $ci(X, Y, \boldsymbol{S}) \geq p_2$ we conclude that $(X \perp\!\!\!\perp Y|\boldsymbol{S})_{\hat{\mathbb{P}}}$. P-values in (p_1, p_2) represent *ambiguous* cases.

2.2 Assumptions

We first make two general assumptions about the data. First, we assume that there is no directed path going from a member of $\boldsymbol{T}$ to a member of $\boldsymbol{V}$. This assumption is biological and states that at a specific generation, the observed phenotypes do not change the DNA of the subjects. Second, we assume that the dataset is not under some collider bias. This implies that if we observe $(X \not\perp\!\!\!\perp Y|\emptyset)_{\hat{\mathbb{P}}}$ then it truly reflects $(X \not\perp\!\!\!\perp Y|\emptyset)_{\mathbb{P}}$ (as the sample sizes goes to infinity).

Low statistical power or small sample size can lead to *partial faithfulness fit*: when multiple unblocked paths exist between an instrument G and a variable Y, some may manifest observed (conditional) association, and some do not. In other words, when $(G \perp\!\!\!\perp Y|\boldsymbol{S})_{\mathcal{G}}$, where $\boldsymbol{S} \subset \boldsymbol{T} \setminus \{Y\}$, then $ci(G, Y, \boldsymbol{S})$ will follow the null distribution. However, if $(G \not\perp\!\!\!\perp Y|\boldsymbol{S})_{\mathcal{G}}$ then $ci(G, Y, \boldsymbol{S})$ may follow some distribution that is too similar to the null distribution. Such partial faithfulness fit is problematic for standard causal discovery algorithms as they heavily rely on a sequential process that uses faithfulness to make decisions. Thus, errors due to partial fit may propagate and distort the output.

While faithfulness may be a reasonable assumption when analyzing variables from $\boldsymbol{T}$, it is problematic when we consider weak instruments. We address this issue by introducing a relaxed assumption about conditional independence between variables from $\boldsymbol{V}$ and $\boldsymbol{T}$, which we denote as *local faithfulness of order k*, for some integer $k \geq 1$. Standard faithfulness dictates that if G is a genetic instrument, $X \in \boldsymbol{T}$ (following the assumptions above), $\boldsymbol{S}, \boldsymbol{S'} \subset \boldsymbol{T}$ are two sets such that $X \notin \boldsymbol{S}$, $X \notin \boldsymbol{S'}$, and we observed $(G \not\perp\!\!\!\perp X|\boldsymbol{S})_{\hat{\mathbb{P}}} \wedge (G \perp\!\!\!\perp X|\boldsymbol{S'})_{\hat{\mathbb{P}}}$ then every path in $\mathcal{G}$ between G and X that is unblocked given S becomes blocked given S'. We alternatively assume that if $|\boldsymbol{S} \setminus \boldsymbol{S'}| \leq k \wedge |\boldsymbol{S'} \setminus \boldsymbol{S}| \leq k$ and we observed $(G \not\perp\!\!\!\perp X|\boldsymbol{S})_{\hat{\mathbb{P}}} \wedge (G \perp\!\!\!\perp X|\boldsymbol{S'})_{\hat{\mathbb{P}}}$ then there **exists a path** π in $\mathcal{G}$ that is unblocked given S but is blocked given S'. In this work we consider $k = 1$ only.

In words, if we observed an association between G and X given a set S such that a small change to S renders G and X independent then the paths between G and X that manifested the association before are now blocked in $\mathcal{G}$ given the new set $\boldsymbol{S}$. Thus, we ease faithfulness in that we do not assume that $(G \perp\!\!\!\perp X|\boldsymbol{S'})_{\hat{\mathbb{P}}} \Rightarrow (G \perp\!\!\!\perp X|\boldsymbol{S'})_{\mathcal{G}}$ necessarily. We alternatively require some local information first that is dependent on observed patterns in the data. Note that the observed association still indicates the existence of an unblocked path between G and X given S, regardless of faithfulness. Given local faithfulness we can now search for evidence for *alternating associations* that have provable properties that can be used for causal discovery.

2.3 cGAUGE: an overview

We first analyze all phenotypes while ignoring the genetic data. This step separates the phenotype pairs into two groups that are each treated differently for extracting causal information. The four steps of the algorithm are as follows:

1. **Single GWAS**: perform a genome-wide association study for each phenotype, adjust for sex and genetic principal components (5). Clump the variants from each phenotype and merge the list.
2. **Skeletons graphs**: identify associations that are robust to conditioning.
3. **Analysis of skeleton non-edges**: analyze phenotype pairs that are unlikely to be under genetic confounding effects, and produce a set of instruments for each pair.
4. **Analysis of skeleton edges**: search for skeleton edges that have evidence for a specific direction hinted by disappearing associations.

For (1) we perform standard association analysis using PLINK [16]. We also avoid an exponential number of statistical tests using practical heuristics. These enables powerful and robust algorithmic strategies. The subsequent analyses are discussed below.

2.4 Skeleton graphs

Inference of a skeleton graph is the initial step that most causal discovery algorithms perform. A *skeleton graph* is an undirected graph $\mathcal{G}$ over a set of observed variables such that an edge $e = (X, Y)$ is added only if there is no evidence that X and Y can be rendered independent by conditioning on a set $\boldsymbol{S}$ (which can be empty) of other observed variables. In other words, skeleton graphs represent associations that are robust to conditioning. Under our assumptions above, these graphs represent a set of candidate pairs that contain the true direct causal links in the underlying causal diagram.

As implied above, the construction of a skeleton depends on a search algorithm that scans through the space of possible sets on which we condition. Naturally, going over all possible sets is not feasible in our case. We deal with this issue using two strategies. First, we limit the number of tested sets per pair of variables to $O(|T|^2)$. Second, we define two skeleton graphs: $\mathcal{G}_T$ and $\mathcal{G}_{V,T}$. $\mathcal{G}_T$ is a standard skeleton inferred by analyzing the phenotypes alone and ignoring the genetic information. $\mathcal{G}_{V,T}$ is a bipartite graph: here we examine the associations between genetic variables and phenotypes.

$\mathcal{G}_T$ is constructed as follows. We start with a complete graph. For each pair $X, Y \in \boldsymbol{T}$ we go over all sets $\boldsymbol{S} \subseteq T \setminus \{X, Y\}$ such that $|\boldsymbol{S}| \leq 2$. For each such $\boldsymbol{S}$ we compute two conditional independence tests: $ci(X, Y, \boldsymbol{S})$ and $ci(X, Y, \boldsymbol{S} \cup \boldsymbol{W})$. If at least of these p-values is $\geq p_1$ then we remove the edge between X and Y from $\mathcal{G}_T$.

$\mathcal{G}_{V,T}$ is constructed as follows. We start with an empty bipartite graph between $\boldsymbol{V}$ and $\boldsymbol{T}$. We then add an edge between a genetic variable G and a phenotype X if the GWAS result marked G as associated with X at a significant level p_1. For each such G, X pair we then go over all sets $\boldsymbol{S} \subseteq T \setminus \{X\}$ such that $|\boldsymbol{S}| \leq 1$. For each such $\boldsymbol{S}$ we compute two conditional independence tests: $ci(X, G, \boldsymbol{S})$ and $ci(X, G, \boldsymbol{S} \cup \boldsymbol{W})$. If at least one of these p-values is $\geq p_2$ then we remove the edge between X and G from $\mathcal{G}_{V,T}$.

In the next section we assume that $\mathcal{G}_{V,T}$ and $\mathcal{G}_T$ correctly reflect the skeletons of the true underlying causal mechanism.

2.5 Analysis of non-edges

Horizontal pleiotropy is a biological phenomenon in which a genetic factor affects two or more biological processes in parallel (and their downstream phenotypes). This poses a fundamental problem for MR methods as it contradicts their basic assumptions. We say that the horizontal pleiotropic confounding is *direct* if the effect of the genetic variable is not mediated by other measured phenotypes in the study (note that additional directed pathways may occur as well). Trait pairs not connected in $\mathcal{G}_T$ have a unique property as explained in the lemma below.

Lemma 2.1. Given the skeleton of the phenotypes $\mathcal{G}_T$ inferred using conditional independence tests among the traits only, a pair $X, Y \in \boldsymbol{T}$ that is disconnected in $\mathcal{G}_T$ cannot have direct (horizontal) pleiotropic effects.

Proof. Had X and Y been directly affected by genetic variables then under the faithfulness assumption there could not have been a conditional independence test to separate them and thus X and Y would have been connected in $\mathcal{G}_T$, a contradiction □

This lemma implies that non-edges in $\mathcal{G}_T$ are good candidates for using MR methods, however

it does not indicate that they are not under any horizontal pleiotropy nor does it tell us which instruments to use. The following results provide practical filters that we call *ImpIV* and *UniqueIV*.

Definition 2.1. Given a directed graph $\mathcal{G} = (\boldsymbol{V}, \boldsymbol{E})$, a pair of nodes $X, Y \in \boldsymbol{V}$, and a set $\boldsymbol{S} \subset \boldsymbol{V}$ such that $X, Y \notin \boldsymbol{S}$ and $(X \perp\!\!\!\perp Y|\boldsymbol{S})_{\mathcal{G}}$ we say that $\boldsymbol{S}$ is a *minimal separating set* of X and Y if there is no subset of it $\boldsymbol{S'} \subset \boldsymbol{S}$ such that $(X \perp\!\!\!\perp Y|\boldsymbol{S'})_{\mathcal{G}}$.

Definition 2.2. Given a directed graph $\mathcal{G} = (\boldsymbol{V}, \boldsymbol{E})$ and a pair of nodes $X, Y \in \boldsymbol{V}$, let $msep(X, Y)$ be the set of all minimal separating sets of X and Y.

Note that these definitions slightly deviate from our notation above as $\boldsymbol{V}$ represents the node set of a general graph and not necessarily our genetic variables. This distinction should be clear from the context.

Definition 2.3. Given $\mathcal{G}_{V,T}$ the true $V - T$ skeleton of the underlying causal diagram $\mathcal{G} = (\boldsymbol{V} \cup \boldsymbol{T}, \boldsymbol{E})$ and $\boldsymbol{X} \subset \boldsymbol{T}$, let $Nei_{sk}(X)$ be the set of all instruments in $\boldsymbol{V}$ that are linked to at least one member of $\boldsymbol{X}$ in $\mathcal{G}_{V,T}$, and let $Nei(X)$ be the set of all instruments in $\boldsymbol{V}$ that are linked to at least one member of $\boldsymbol{X}$ by an unblocked pathway in $\mathcal{G}$ (i.e., when conditioning on $\boldsymbol{S} = \emptyset$).

Theorem 2.1. (ImpIV) Given a pair of phenotypes $X, Y \in \boldsymbol{T}$ not adjacent in the true skeleton $\mathcal{G}_T$ and $(X \not\perp\!\!\!\perp Y|\emptyset)_{\mathcal{G}}$, the variants in $Nei_{sk}(msep(X, Y))$ are not proper instruments relative to (X, Y).

Proof. X and Y are not linked in $\mathcal{G}_T$ and $(X \not\perp\!\!\!\perp Y|\emptyset)_{\mathcal{G}}$, thus $msep(X, Y)$ contains at least one non-empty set required for blocking the active paths between X and Y (i.e., active paths when conditioning on $\emptyset$). Let $\boldsymbol{S}$ be such a minimal separating set. To prove the theorem it is enough to show that every member of $\boldsymbol{S}$ has at least one active path to Y (conditioned on $\emptyset$) that does not contain X.

Note that by definition, any $U \in \boldsymbol{S}$ cannot be a collider on all paths between X and Y that contain it, as otherwise it can be removed from $\boldsymbol{S}$, contradicting $\boldsymbol{S}$ being a minimal separating set. Thus, U has an outgoing edge in at least one path between X and Y. If at least one of these paths is active when conditioned on $\emptyset$ then we are done. Otherwise, all of the paths with an outgoing edge from U are blocked (conditioned on $\emptyset$) and therefore must have at least one collider. Thus, there must exist a path p in which U has an outgoing edge and all colliders are members of $\boldsymbol{S}$. If all colliders appear in the subpath between X and U, then again we are done. Otherwise, let V_1 be the closest collider to U in the subpath of p from U to Y. As $V_1 \in \boldsymbol{S}$, then by the same arguments above it either (1) blocks an active path between X and Y (conditioned on $\emptyset$), or (2) has a path p_1 in which it has an outgoing edge and all colliders are members of $\boldsymbol{S}$ and at least one of them is in the subpath of p_1 between V_1 and Y. If (1) occurs then we are done as we can now concatenate U's collider-free path to V_1 with the

active path between V_1 and Y. Otherwise, we can now create an active path (conditioned on $\emptyset$) between U and V_1, and we are left with showing that V_1 has an active path to Y. By the same argument about p, p_1 has a collider V_2 on its subpath from V_1 to Y.

We can now repeat the arguments that we used above for $U = V_0$ and V_1 for a new node V_2 and repeat the process to create a series of nodes $V_1, V_2, \ldots, V_k$, such that there is an active path between U and V_1 and between each V_i to V_{i+1}. However, we need to show that there is a way to construct this series to be acyclic and therefore final. Indeed, if at some point in the process of creating the series we try to add a new collider V_i in a path p_i that we have seen before and p_i has no additional colliders between V_{i-1} and Y, then again we are done as we now have an active path from U to Y through $V_1, \ldots, V_{i-1}$. Otherwise, we update V_i to be the next new collider in the path. As the series is either terminated or extended by a new node it is final and must end, and so does our constructed active path from U to Y that is not through X $\square$

The theorem above provides a way to remove genetic variables that are not instruments relative to X, Y. However, the implied criterion does not cover all improper instruments as we prove below.

Lemma 2.2. Given a pair of phenotypes $X, Y \in \boldsymbol{T}$ not adjacent in $\mathcal{G}_T$ and $(X \not\perp\!\!\!\perp Y|\emptyset)_{\mathcal{G}}$, the variant set $Nei(\{X\}) \setminus Nei_{sk}(msep(X,Y))$ may contain improper instruments relative to (X, Y)

Proof. We prove this by example. Consider a simple graph with a single genetic variable G and four phenotypes: X, Y, V_1, V_2. The phenotype graph has the following three paths: $U \rightarrow V_1 \rightarrow X$, $U \rightarrow V_2 \rightarrow Y$, and $X \rightarrow U \leftarrow Y$. In addition we have the edge $G \rightarrow U$. Note that in our example, X and Y are separated only via the set $\{V_1, V_2\}$. Thus, U, which is connected to G, is not a member of any minimal separating set. Moreover, $(X \not\perp\!\!\!\perp G|\emptyset)_{\mathcal{G}}$, and $(Y \not\perp\!\!\!\perp G|\emptyset)_{\mathcal{G}}$, implying that G is a member of $Nei(\{X\})$. $\square$

Definition 2.4. Given the bipartite skeleton $\mathcal{G}_{V,T}$ and $X \in \boldsymbol{T}$, let $Nei_1(X)$ be the set of all neighbors of X that are not linked to any other member of $\boldsymbol{T}$.

Theorem 2.2. (UniqueIV) Given a pair of phenotypes $X, Y \in \boldsymbol{T}$ not adjacent in $\mathcal{G}_T$, and such that $|Nei_1(X)| > 0$, the variants in $Nei_1(X)$ are proper instruments relative to (X, Y).

Proof. By assumption about the correctness of $\mathcal{G}_{V,T}$, members of $Nei_1(X)$ cannot have outgoing edges to members of $\boldsymbol{T}$ other than X. Similarly, there cannot be an unobserved variable U (a confounder) that has paths going to both a member of $Nei_1(X)$ and Y. Finally, by our assumption that genetic variants cannot be affected by members of $\boldsymbol{T}$, there cannot be either directed pathways from X or Y to members of $Nei_1(X)$, nor is there another member $Z \in \boldsymbol{T}$ that affects X or Y and is a member of $Nei_1(X)$. $\square$

To summarize, the two theorems in this section provide practical methods to either remove improper instruments or to select a potentially small set of proper instruments. The trade-off between the two approaches is that the first one is likely to result in larger instrument set sizes, but without the guarantees of the second approach. If the resulting set indeed contains only proper instruments, then the first approach will likely have greater statistical power as compared to the second approach as it will contain proper instruments whose effect on X may be mediated by other observed variables.

2.6 Analysis of skeleton edges

In the analysis above we excluded the detected edges in $\mathcal{G}_T$. These edges are a mixture of (by assumption) a few errors, confounding effects, and direct causal links. We next discuss situations in which the conditional independence observed relative to skeleton edges can be used as evidence for identifying causal direction.

Theorem 2.3. (ExSep) Let G be a genetic variable and (X, Y) be an edge in G_T. If $(G \not\perp\!\!\!\perp Y|\emptyset)_{\hat{\mathbb{P}}}$, and $(G \perp\!\!\!\perp Y|X)_{\hat{\mathbb{P}}}$, then under the assumption of local faithfulness and no collider bias of the input dataset there exists a directed path from X to Y.

Proof. By the assumptions and the observed dependence $(G \not\perp\!\!\!\perp Y|\emptyset)_{\hat{\mathbb{P}}}$ there is a path p_Y from G to Y that has no colliders, has X with an outgoing edge (to create the independence $(G \perp\!\!\!\perp Y|X)_{\hat{\mathbb{P}}}$), and there is no directed path from X into G. Thus, the subpath from X to Y must be a directed path without $\leftrightarrow$ edges (i.e., confounded by a common cause) or an outgoing edge from Y, as both of these imply the existence of a collider on the subpath between X and Y that cannot explain the newly created independence when conditioning on X. $\square$

In the proof above we used the local faithfulness assumption. Practically, since we already observed $(G \not\perp\!\!\!\perp X|\emptyset)_{\hat{\mathbb{P}}}$ and $(G \not\perp\!\!\!\perp Y|\emptyset)_{\hat{\mathbb{P}}}$ we can reasonably assume that there is enough detection power for associations in the examined local area of the causal diagram and thus use local faithfulness as evidence for a blockage of a path. In addition, note that we did not make any assumptions about G (e.g., $G \in Nei_1(X)$). This is used in the next section to develop a statistical test for identifying graphical patterns with the properties above. Finally, in practice we check if $(G \not\perp\!\!\!\perp X|\boldsymbol{W})_{\hat{\mathbb{P}}}$, $(G \not\perp\!\!\!\perp Y|\boldsymbol{W})_{\hat{\mathbb{P}}}$, and $(G \perp\!\!\!\perp Y|\{X\} \cup \boldsymbol{W})_{\hat{\mathbb{P}}}$, as by assumption $\boldsymbol{W}$ are exogenous (the proof remains similar).

3 Empirical Bayes analysis

Using **Theorem 2.3** in practice requires identifying cases with $(G \not\perp\!\!\!\perp Y|\emptyset)_{\hat{\mathbb{P}}}$ and $(G \perp\!\!\!\perp Y|X)_{\hat{\mathbb{P}}}$ in real data, which we call *ExSep* (Exposure-base Separation) events in the main text. In

this section we utilize the *two-groups* empirical Bayes framework to develop statistical tests with a null hypothesis that no such cases exist for a given X and Y. We start with a brief introduction of the two-groups model, for a full description and background see [17].

Given a large set of N hypotheses tested in a large-scale study, the two-groups model provides a simple Bayesian framework for multiple testing: each of the N cases (e.g., genes in a gene expression study) are either null or non-null with prior probability π_0 and $\pi_1 = 1 - \pi_0$, and with z-scores (or p-values) having density either $f_0(z)$ or $f_1(z)$. When the assumptions of the statistical test are satisfied, f_0 is a standard normal (or a uniform distribution for p-values), and is called the *theoretical null*. However, the Bayesian framework can be used to infer the parameters of f_0 from the data, as they can be slightly different than those of the theoretical null [17]. The mixture density and probability distributions are:

$$f(z) = \pi_0 f_0(z) + \pi_1 f_1(z)$$
$$F(z) = \pi_0 F_0(z) + \pi_1 F_1(z)$$

For a rejection area $\mathbf{Z}_y = (-\infty, y)$, using Bayes rule we get:

$$Fdr(\mathbf{Z}_y) \equiv Pr\{null|z \in \mathbf{Z}_y\} = \pi_0 F_0(y)/F(y)$$

We call Fdr the *(Bayes) false discovery rate* for $\mathbf{Z}_y$: this is the probability we would make a false discovery if we report $\mathbf{Z}_y$ as non-null. The Bayesian framework of the model allows us to define a local version of the FDR. That is, if $\mathbf{Z}_y$ is a single point z_0 we define the *local (Bayes) false discovery rate* as:

$$fdr(z_0) \equiv Pr\{null|z = z_0\} = \pi_0 f_0(z_0)/f(z_0)$$

Moreover, we can also define the *local (Bayes) true discovery rate* as:

$$tdr(z_0) \equiv Pr\{non - null|z = z_0\} = 1 - fdr(z_0)$$

3.1 Problem definition

In the first step of cGAUGE we perform a GWAS analysis: we quantify the association between all genetic variables in $\boldsymbol{V}$ and $Y \in \boldsymbol{T}$, adjusting for the set of exogenous variables $\boldsymbol{W}$. This step produces a set of $|\boldsymbol{V}|$ p-values that we can transform to $|\boldsymbol{V}|$ z-scores, denoted as $z_1 = (z_1^1, z_1^2, \ldots, z_1^{|\boldsymbol{V}|})$. However, to test for the existence of the ExSep graphical patterns (of **Theorem 2.3**), we repeat the analysis, adding X to the set of variables that are adjusted for. This analysis produces another vector of z-scores $z_2 = (z_2^1, z_2^2, \ldots, z_2^{|\boldsymbol{V}|})$. We assume that z_1^i and z_2^i each follows a two-groups mixture model as explained above. However, we do not know their joint distribution.

Let h_1 and h_2 be the binary vectors that represent the true unknown group assignments for each genetic variable, in each of the tests above. Using the two-groups notation, the ExSep graphical pattern can be read as follows: there exists a genetic variable G with $h_1^G = 1$ and $h_2^G = 0$. Thus, under the null hypothesis that there are no such patterns: $(h_1^i = 1) \Rightarrow (h_2^1 = 1)$.

3.2 ExSep: Model selection based LRT

We start with a simplifying assumption that the two groups models of z_1 and z_2 are mixtures of two normal distributions. Similar models were explored in [18, 19], and were shown to provide a fast and robust approximation. Thus, we assume that f_0 is a normal distribution $\mathcal{N}(\mu_0, \sigma_0)$ and f_1 is another normal distribution $\mathcal{N}(\mu_1, \sigma_1)$. Inference of the parameters $\mu_0, \mu_1, \sigma_0, \sigma_1, \pi_0$ is done using the *znormix* Expectation-Maximization (EM) algorithm, as explained in [18, 19]. We do not set constraints for f_0 to follow the theoretical null, but set $\mu_1 > 3$ as we expect genetic data to have the non-null p-values at a very significant level (e.g., $p < 0.001$).

We fit a two-groups mixture model for z_1 and z_2 separately. We denote the parameters for z_1 as $\theta_1 = (\pi_{1,0}, \mu_{1,0}, \mu_{1,1}, \sigma_{1,0}, \sigma_{1,1})$ and the parameters for z_2 as $\theta_2 = (\pi_{2,0}, \mu_{2,0}, \mu_{2,1}, \sigma_{2,0}, \sigma_{2,1})$. We assume for the rest of this section that the means (all μ parameters) and the variances are estimated correctly and hold them fixed. We now move on to model joint distribution of (z_1, z_2) which is a mixture of up to four bivariate Gaussian distributions:

$$\mathcal{N}_1 : N\left(\begin{pmatrix}\mu_{1,0}\\ \mu_{2,0}\end{pmatrix}, \begin{pmatrix}\sigma_{1,0}^2 & \rho_{0,0}\sigma_{1,0}\sigma_{2,0}\\ \rho_{0,0}\sigma_{1,0}\sigma_{2,0} & \sigma_{2,0}^2\end{pmatrix}\right)$$
$$\mathcal{N}_2 : N\left(\begin{pmatrix}\mu_{1,0}\\ \mu_{2,1}\end{pmatrix}, \begin{pmatrix}\sigma_{1,0}^2 & \rho_{0,1}\sigma_{1,0}\sigma_{2,1}\\ \rho_{1,0}\sigma_{1,0}\sigma_{2,1} & \sigma_{2,1}^2\end{pmatrix}\right)$$
$$\mathcal{N}_3 : N\left(\begin{pmatrix}\mu_{1,1}\\ \mu_{2,0}\end{pmatrix}, \begin{pmatrix}\sigma_{1,1}^2 & \rho_{1,0}\sigma_{1,1}\sigma_{2,0}\\ \rho_{1,0}\sigma_{1,1}\sigma_{2,0} & \sigma_{2,0}^2\end{pmatrix}\right)$$
$$\mathcal{N}_4 : N\left(\begin{pmatrix}\mu_{1,1}\\ \mu_{2,1}\end{pmatrix}, \begin{pmatrix}\sigma_{1,1}^2 & \rho_{1,1}\sigma_{1,1}\sigma_{2,1}\\ \rho_{1,1}\sigma_{1,1}\sigma_{2,1} & \sigma_{2,1}^2\end{pmatrix}\right)$$

$\mathcal{N}_1$ is the density of genetic variables that are null in both z_1 and z_2, that is, cases for which $h_1^i = 0 \wedge h_2^i = 0$. Similarly, $\mathcal{N}_2$ represents cases for which $h_1^i = 0 \wedge h_2^i = 1$, $\mathcal{N}_3$ represents cases for which $h_1^i = 1 \wedge h_2^i = 0$, and $\mathcal{N}_4$ represents cases for which $h_1^i = 1 \wedge h_2^i = 1$. Under our simplifying assumptions the unknown parameters of the model above are the correlation parameters ρ of each bivariate distribution and the prior of each of the four densities.

We estimate these parameters using a simple grid search over the four ρ parameters, testing all possible combinations along a four dimensional grid with values $0, 0.1, 0.2, \ldots, 0.9$. For each combination, all parameters above are fixed and we can therefore estimate both the priors and

the likelihood of the model using a simple variation of the EM algorithm.

Under the null hypothesis z' can be modeled as a mixture of $\mathcal{N}_1, \mathcal{N}_2$, and $\mathcal{N}_4$, without the $\mathcal{N}_3$ density component. We therefore utilize the same grid-search algorithm as above, this time ignoring the $\mathcal{N}_3$ cluster. The log-likelihood from the full model l and the log-likelihood from the null model l_0 are then used for a likelihood ratio test with the statistic $\lambda = -2(l_0 - l) \sim \chi^2_2$ (under the null hypothesis).

Quantifying the improvement in fit as a function of the number of clusters is a standard approach in model selection. However, in the general case, a likelihood ratio test approximation that relies on EM-based estimates may not approximate the true null hypothesis well. Our grid search approach is faster and robust as it searches through the entire space of potential within distribution correlations, and apply the EM in a very specific case where all parameters but the prior are hold fixed. Future studies can try to improve this method using parametric bootstrap to compute conservative empirical p-values. However, this approach is slow and requires many bootstrap repeats to achieve reasonable estimates that will survive adjustment for multiple testing. Moreover, parametric bootstrap tests are very sensitive to errors in the estimation of the true null distribution.

3.3 As an additional score for MR analysis

For each analyzed phenotype pair, we used the two-groups model to examine the p-value distribution of the variants of the exposure with the outcome. Note that for inference of causal diagrams alone, instruments can point out potential flow of causal information without necessarily trying to estimate effects using a parametric model as MR methods do. In theory, under faithfulness, if X is a cause of Y then all instruments of X have an unblocked path to Y and should therefore be statistically associated with it. Thus, we can simply ask if a set of instruments relative to X, Y is statistically associated with Y in a consistent way. We quantify this by computing the proportion of non-null p-values, using the local FDR method implemented in the limma R package as it is very fast and unlike znormix can also handle a small set of instruments [20, 21].

4 Simulations

4.1 Full causal models

We generated random causal graphs with $p = 15$ nodes as follows. Let B represent the adjacency matrix of a randomly generated graph. For each possible directed edge (the off-

diagonal entries in B), we added its entry in B to the set of edges with probability d/p, where d marks the expected outgoing degree of each node. Then, for each edge we set its non-zero entry in B by sampling from a uniform distribution $U[(-0.95. - 0.1] \cup (0.1, 0.95)]$. We then added a set of causal instruments for each node and extended B to hold both the traits and the instruments. The number of instruments per node was selected from a uniform discrete distribution $U[10, 20]$. At this step B contains a causal graph between 15 traits and a set of instruments for each node, such that each instrument is directly linked to a single node. For each instrument, we then flip a "coin" with probability p_{pleio} to decide if it will be linked to other traits as well. If the result is tails, then we add direct edges from the instrument to a set of randomly selected traits, with the set size selected from a uniform distribution $U[1, 10]$. We tested different combinations of d and p_{pleio} to simulate graphs of different complexity: $d \in \{1, 1.25, 1.5, 1.75, 2\}$, and $p_{pleio} \in \{0, 0.1, 0.2, 0.3, 0.4\}$.

Given the graph above B, we move now to generate the data over $N = 2000$ samples. We used the linear SEM framework to simulate

$$X_i = \sum_{j=1}^{nrow(B)} B_{i,j} X_j + \epsilon_i$$

where $nrow(B)$ is the total number of nodes in B (instruments and traits), and $\epsilon_i \sim \mathcal{N}(0, 1)$. Note that all instruments are exogenous and have no incoming arrows. Thus, when simulating a single sample, each instrument was generated by sampling a random binary vector with probability p_{maf} selected from a uniform distribution $U[0.05, 0.4]$ (set for each instrument before starting to simulate the samples). As we are simulating from a linear SEM, using the exogenous variables, the ϵ variables, and B to generate samples is trivial as the data vector of a sample X satisfies:

$$X = (I - B)^{-1} e$$

where e represents all exogenous values randomly selected for the sample (e.g., instruments and ϵ variables). Note that this simulation step is valid even if B contains cycles, see the introduction for more details on cycles. Also, note that for non-linear SEMs Fisher's fixed point method can be used to simulate data, but we did not explore this approach in this work [22].

To run the cGAUGE algorithms we repeated the simulations above and tested different values of p_1 and p_2: $p_1 \in \{0.01, 0.001.10^{-04}, 10^{-05}\}$ and $p_2 = p_1, 10 * p_1, 100 * p_1$. Combined with the different combinations of d and p_{pleio} above, for each combination of d, p_{pleio}, p_1, p_2 we generated 40 different graphs with their synthetic datasets. This amounted to $12,000$ synthetic datasets on which we tested our methods and others.

4.2 Testing local faithfulness and MR failures

We tested the simple graph structures of **Figure 2** in the main text to illustrate the limitations of MR methods when latent confounders have many genetic instruments and to illustrate the local faithfulness assumption. For simplicity, we use the simple notation from the main text: let G be a binary genetic variable, X, Y two observed continuous variables, and U an unobserved continuous variable. We tested three cases in which all genetic variables were linked to a single non-genetic variable. In the first case (denoted as $\mathcal{C}_1$), we had 30 genetic variables, 20 that directly affect U (denoted as $G_{u,j}$, $j = 1, \ldots, 20$), and 10 that directly affect X (denoted as $G_{x,j}$, $j = 1, \ldots, 10$). In the second and third cases (denoted as $\mathcal{C}_2$,and $\mathcal{C}_3$ respectively) we had a single genetic variable connected only to X.

All three cases had U as a common cause of X and Y but differed in the relationships among X, Y:

$$\mathcal{C}_1 : X \not\rightarrow Y \wedge Y \not\rightarrow X$$
$$\mathcal{C}_2 : X \rightarrow Y \wedge Y \not\rightarrow X$$
$$\mathcal{C}_3 : X \rightarrow Y \wedge Y \rightarrow X$$

All simulated cases followed linear relationships between the variables as explained above.

Datasets were simulated using standard normal errors for continuous variables and sampling G from a Bernoulli distribution with a different probability to simulate different minor allele frequencies. Then, each edge had a coefficient that determined its added value in the structural equation. For example, the model of $\mathcal{C}_3$ can be written:

$$u = \epsilon_u$$
$$g = \epsilon_g$$
$$x = \alpha_1 g + \alpha_3 u + \epsilon_x$$
$$y = \alpha_2 x + \alpha_3 u + \epsilon_y$$

where $\epsilon_g \sim Bernoulli(p)$, and $\epsilon_u, \epsilon_x, \epsilon_y \sim N(0, 1)$ i.i.d.

For $\mathcal{C}_1$ we sampled p and the causal quantities as explained in the previous section. In the other cases we used $p = 0.05$ to simulated relatively rare instruments and set $\alpha_2 = \alpha_3 = 0.5$. For each graph, we simulated 100 datasets with 2,000 samples. Conditional independence was tested using generalized likelihood tests via either linear regression or logistic regression (e.g., if G was considered as the dependent variable).

For $\mathcal{C}_2$ and $\mathcal{C}_3$ we were interested in the performance of conditional independence tests and their fit with the theory. We computed the frequencies of mixed results for each case. That is,

we compared how many instruments had: (1) disappearing correlations - $(G \not\perp\!\!\!\perp X|\emptyset)_{\hat{\mathbb{P}}_{sim}}$, $(G \not\perp\!\!\!\perp Y|\emptyset)_{\hat{\mathbb{P}}_{sim}}$, and $(G \perp\!\!\!\perp Y|X)_{\hat{\mathbb{P}}_{sim}}$, (2) emerging correlations - $(G \not\perp\!\!\!\perp X|\emptyset)_{\hat{\mathbb{P}}_{sim}}$, $(G \perp\!\!\!\perp Y|\emptyset)_{\hat{\mathbb{P}}_{sim}}$, and $(G \not\perp\!\!\!\perp Y|X)_{\hat{\mathbb{P}}_{sim}}$, and (3) consistent correlations - $(G \not\perp\!\!\!\perp X|\emptyset)_{\hat{\mathbb{P}}_{sim}}$, $(G \not\perp\!\!\!\perp Y|\emptyset)_{\hat{\mathbb{P}}_{sim}}$, and $(G \not\perp\!\!\!\perp Y|X)_{\hat{\mathbb{P}}_{sim}}$. Here, $\hat{\mathbb{P}}_{sim}$, is the empirical distribution of a single simulated dataset.

References

[1] Pearl, J. *Causality: Models, Reasoning and Inference* (Cambridge University Press, New York, NY, USA, 2009), 2nd edn.

[2] Spirtes, P., Glymour, C. & Scheines, R. *Causation, Prediction, and Search, 2nd Edition*, vol. 1 of *MIT Press Books* (The MIT Press, 2001).

[3] Pearl, J. & Dechter, R. Identifying Independencies in Causal Graphs with Feedback. In *UAI'96 Proceedings of the Twelfth international conference on Uncertainty in artificial intelligence*, 2, 420–426 (1996). arXiv:1011.1669v3.

[4] Spirtes, P. Directed cyclic graphical representations of feedback models. In *Proceedings of the Eleventh Conference Annual Conference on Uncertainty in Artificial Intelligence (UAI-95)*, 491–498 (Morgan Kaufmann, San Francisco, CA, 1995).

[5] Neal, R. M. On Deducing Conditional Independence from d-Separation in Causal Graphs with Feedback. *Journal of Artificial Intelligence Research* **12**, 87 (2000). 1106.0237v1.

[6] Poole, D. & Crowley, M. Cyclic causal models with discrete variables: Markov chain equilibrium semantics and sample ordering. In *IJCAI International Joint Conference on Artificial Intelligence*, 1060–1068 (2013).

[7] Zhang, J. On the completeness of orientation rules for causal discovery in the presence of latent confounders and selection bias. *Artificial Intelligence* **172**, 1873–1896 (2008).

[8] Richardson, T. S. A Discovery Algorithm for Directed Cyclic Graphs. In *UAI'96 Proceedings of the Twelfth international conference on Uncertainty in artificial intelligence*, 2, 454–461 (1996). 1302.3599.

[9] Hyttinen, A., Hoyer, P. O., Eberhardt, F. & Järvisalo, M. Discovering Cyclic Causal Models with Latent Variables: A General {SAT}-Based Procedure. In *Proceedings of UAI*, 301–310 (2013).

[10] Li, J. & Wang, Z. J. Controlling the False Discovery Rate of the Association/Causality Structure Learned with the {PC} Algorithm. *Journal of Machine Learning Research* **10**, 475–514 (2009).

[11] Strobl, E. V. & Spirtes, P. L. Estimating and Controlling the False Discovery Rate for the PC Algorithm Using Edge-Specific P-Values. *arXiv:1702.03877v2 [stat.ME]* (2016). URL http://arxiv.org/abs/1607.03975. arXiv:1607.03975v1.

[12] Wright, S. The Method of Path Coefficients. *The Annals of Mathematical Statistics* **5**, 161–215 (1934). URL http://projecteuclid.org/euclid.aoms/1177732676. aoms/1177732676.

[13] Bowden, R. J. & Turkington, D. A. *Instrumental variables*, vol. 8 (Cambridge University Press, 1990).

[14] Pingault, J. *et al.* Using genetic data to strengthen causal inference in observational research. *NATURE REVIEWS GENETICS* **19**, 566–580 (2018).

[15] O'Connor, L. J. & Price, A. L. Distinguishing genetic correlation from causation across 52 diseases and complex traits (2018).

[16] Chang, C. C. *et al.* Second-generation PLINK: Rising to the challenge of larger and richer datasets. *GigaScience* **4** (2015). 1410.4803.

[17] Efron, B. *Large-scale inference : Empirical Bayes methods for estimation, testing, and prediction* (Cambridge University Press, 2010).

[18] McLachlan, G., Bean, R. & Jones, L. B.-T. A simple implementation of a normal mixture approach to differential gene expression in multiclass microarrays. *Bioinformatics* **22**, 1608–1615 (2006). URL https://doi.org/10.1093/bioinformatics/btl148. http://oup.prod.sis.lan/bioinformatics/article-pdf/22/13/1608/483495/btl148.pdf.

[19] Amar, D., Shamir, R. & Yekutieli, D. Extracting replicable associations across multiple studies: Empirical Bayes algorithms for controlling the false discovery rate. *PLOS Computational Biology* **13**, e1005700 (2017). URL https://doi.org/10.1371/journal.pcbi.1005700.

[20] Ritchie, M. E. *et al.* Limma powers differential expression analyses for RNA-sequencing and microarray studies. *Nucleic Acids Research* **43**, e47 (2015).

[21] Langaas, M., Lindqvist, B. H. & Ferkingstad, E. Estimating the proportion of true null hypotheses, with application to DNA microarray data. *Journal of the Royal Statistical Society. Series B: Statistical Methodology* **67**, 555–572 (2005). 1206.1874.

[22] Fisher, F. A Correspondence Principle for Simultaneous Equation Models. *Econometrica: Journal of the Econometric Society* **38**, 73–92 (1970). URL http://www.jstor.org/stable/10.2307/1909242.
