# Supplementary material for: Graphical analysis for phenome-wide causal discovery in genotyped population-scale biobanks
Source: Nat Commun. 2021 Jan 13;12:350. doi: 10.1038/s41467-020-20516-2 (PMC7806647; doi:10.1038/s41467-020-20516-2)
Supplement: Supplementary file 2 — Reporting Summary [file 41467_2020_20516_MOESM2_ESM.pdf]

nature research

Corresponding author(s): Manuel A. Rivas

Last updated by author(s): 11/19/2020

Reporting Summary

Nature Research wishes to improve the reproducibility of the work that we publish. This form provides structure for consistency and transparency in reporting. For further information on Nature Research policies, seeAuthors & Referees and theEditorial Policy Checklist .

Statistics

For all statistical analyses, confirm that the following items are present in the figure legend, table legend, main text, or Methods section.

n/a	Confirmed	
☐	☒	The exact sample size (*n*) for each experimental group/condition, given as a discrete number and unit of measurement
☐	☒	A statement on whether measurements were taken from distinct samples or whether the same sample was measured repeatedly
☐	☒	The statistical test(s) used AND whether they are one- or two-sided<br>*Only common tests should be described solely by name; describe more complex techniques in the Methods section.*
☐	☒	A description of all covariates tested
☐	☒	A description of any assumptions or corrections, such as tests of normality and adjustment for multiple comparisons
☐	☒	A full description of the statistical parameters including central tendency (e.g. means) or other basic estimates (e.g. regression coefficient) AND variation (e.g. standard deviation) or associated estimates of uncertainty (e.g. confidence intervals)
☐	☒	For null hypothesis testing, the test statistic (e.g. *F*, *t*, *r*) with confidence intervals, effect sizes, degrees of freedom and *P* value noted<br>*Give P values as exact values whenever suitable.*
☒	☐	For Bayesian analysis, information on the choice of priors and Markov chain Monte Carlo settings
☒	☐	For hierarchical and complex designs, identification of the appropriate level for tests and full reporting of outcomes
☐	☒	Estimates of effect sizes (e.g. Cohen's *d*, Pearson's *r*), indicating how they were calculated

Our web collection on statistics for biologists contains articles on many of the points above.

Software and code

Policy information about availability of computer code

Data collection	Data was retrieved from the UK-Biobank using application 24983. Summary statistics were computed using PLINK (PLINK v2.00a2LM 64-bit Intel (20 Feb 2018)).
Data analysis	The R code for the new method is available at https://github.com/david-dd-amar/cGAUGE/. This page also provides the code for preprocessing UK-Biobank data via PLINK. Code for the preprocessing of UK Biobank biomarker data is available at https://github.com/rivas-lab/public-resources/tree/master/uk_biobank/laboratory-tests. The code was tested in R version 3.5.1. We also use the following R packages: MendelianRandomization (version 0.4.2), limma (version 3.42.2), bnlearn (version 4.5), MRPRESSO (version 1.0). We used Cytoscape version 3.7.2 for network visualization and clustering.

For manuscripts utilizing custom algorithms or software that are central to the research but not yet described in published literature, software must be made available to editors/reviewers. We strongly encourage code deposition in a community repository (e.g. GitHub). See the Nature Research guidelines for submitting code & software for further information.

Data

Policy information about availability of data

All manuscripts must include a data availability statement. This statement should provide the following information, where applicable:

- Accession codes, unique identifiers, or web links for publicly available datasets
- A list of figures that have associated raw data
- A description of any restrictions on data availability

Data was retrieved from the UK-Biobank using application 24983.

Field-specific reporting

Please select the one below that is the best fit for your research. If you are not sure, read the appropriate sections before making your selection.

- [x] Life sciences
- [ ] Behavioural & social sciences
- [ ] Ecological, evolutionary & environmental sciences

For a reference copy of the document with all sections, see nature.com/documents/nr-reporting-summary-flat.pdf

Life sciences study design

All studies must disclose on these points even when the disclosure is negative.

Sample size	We analyzed data from 337,198 subjects from the UK Biobank. We used the data for computing variant-trait and trait-trait associations (with and without conditioning on other variable sets). Although no power calculation was performed in advance, this sample size is sufficient for identifying associations among the variables discussed in this paper. Moreover, this dataset is the largest publicly available phenome-wide observational data available at the time of the preparation of the paper and was (and still is) used in multiple studies that considered traits and genetic variants that are even more rare than those analyzed in this paper.
Data exclusions	The 337, 198 subset of UK Biobank subjects were selected using the quality assurance and relative filtering presented in DeBoever et al. 2019 (https://doi.org/10.1038/s41467-018-03910-9). Briefly, low quality and non EUR British ancestry samples were excluded. These are well established preprocessing steps for cleaning genetic data before association analysis.
Replication	not applicable for this study: we presented a novel computational method and reported its finding in the UK Biobank.
Randomization	not applicable for this study: we present novel methods for extracting causal information from observational data, which by definition is not randomized.
Blinding	not applicable for this study: we present novel methods for extracting causal information from existing unblinded observational data.

Reporting for specific materials, systems and methods

We require information from authors about some types of materials, experimental systems and methods used in many studies. Here, indicate whether each material, system or method listed is relevant to your study. If you are not sure if a list item applies to your research, read the appropriate section before selecting a response.

Materials & experimental systems

n/a	Involved in the study	
x		Antibodies
x		Eukaryotic cell lines
x		Palaeontology
x		Animals and other organisms
x		Human research participants
x		Clinical data

Methods

n/a	Involved in the study	
x		ChIP-seq
x		Flow cytometry
x		MRI-based neuroimaging
