# Supplementary material for: Graphical analysis for phenome-wide causal discovery in genotyped population-scale biobanks
Source: Nat Commun. 2021 Jan 13;12:350. doi: 10.1038/s41467-020-20516-2 (PMC7806647; doi:10.1038/s41467-020-20516-2)
Supplement: Supplementary file 3 — Description of Additional Supplementary Files [file 41467_2020_20516_MOESM3_ESM.pdf]

Description of Additional Supplementary Files

Supplementary Data 1: Empirical FDRs of the MR and the naive count ExSep methods

Supplementary Data 2: Number of discoveries of the MR and the naive count ExSep methods

Supplementary Data 3: Empirical FDRs of the ExSep model selection test

Supplementary Data 4: Number of discoveries of the ExSep model selection test

Supplementary Data 5: Empirical FDRs and FPRs using the $\square_1$ estimates

Supplementary Data 6: Number of discoveries using the $\square_1$ estimates ($\square_1$ >thr)

Supplementary Data 7: Comparison with CAUSE: FDR and number of discoveries (c-MRPRESSO and c-IVW: utilizing the MR methods with a cGAUGE UniqueIV filter, (*) a variation of cGAUGE with p1=1e-05 and p2=0.001, otherwise cGAUGE was run with p1=0.001, and p2=0.01).

Supplementary Data 8: The phenotypes analyzed in the UKBB data

Supplementary Data 9: Selected MR results after the uniqueIV filter, with 0.1 FDR adjustment and pi1>0.25 (MS_test: p-value for the ExSep model-selection test, p_ivw: IVW p-value for causal effect, p_het_ivw: IVW p-value for effect heterogeneity, p_mrpresso: MRPRESSO p-value for causal effect, mrepresso_global: MRPRESSO p-value for the global test, qvalue: FDR adjusted q-values).

Supplementary Data 10: Significant ExSep model selection test results (0.01 FDR) that are not in the MR results (MS_test: p-value for the ExSep model-selection test, p_ivw: IVW p-value for causal effect, p_het_ivw: IVW p-value for effect heterogeneity, p_mrpresso: MRPRESSO p-value for causal effect, mrepresso_global: MRPRESSO p-value for the global test, qvalue: FDR adjusted q-values).

Supplementary Data 11: All causal inference results after the uniqueIV filter with $p_1=10^{-06}$ and $p_2=0.01$ (MS_test: p-value for the ExSep model-selection test, p_ivw: IVW p-value for causal effect, p_het_ivw: IVW p-value for effect heterogeneity, p_mrpresso: MRPRESSO p-value for causal effect, mrepresso_global: MRPRESSO p-value for the global test, qvalue: FDR adjusted q-values).

Supplementary Data 12: All causal inference results after the uniqueIV filter with $p_1=10^{-06}$ and $p_2=0.001$ (MS_test: p-value for the ExSep model-selection test, p_ivw: IVW p-value for causal effect, p_het_ivw: IVW p-value for effect heterogeneity, p_mrpresso: MRPRESSO p-value for causal effect, mrepresso_global: MRPRESSO p-value for the global test, qvalue: FDR adjusted q-values).

Supplementary Data 13: All causal inference results after the uniqueIV filter with $p_1=10^{-07}$ and $p_2=0.01$ (MS_test: p-value for the ExSep model-selection test, p_ivw: IVW p-value for causal effect, p_het_ivw: IVW p-value for effect heterogeneity, p_mrpresso: MRPRESSO p-value for causal effect, mrepresso_global: MRPRESSO p-value for the global test, qvalue: FDR adjusted q-values).

Supplementary Data 14: All causal inference results after the uniqueIV filter with $p_1=10^{-07}$ and $p_2=0.001$ (MS_test: p-value for the ExSep model-selection test, p_ivw: IVW p-value for causal effect, p_het_ivw: IVW p-value for effect heterogeneity, p_mrpresso: MRPRESSO p-value for causal effect, mrepresso_global: MRPRESSO p-value for the global test, qvalue: FDR adjusted q-values).

Supplementary Data 15: The instrument sets discovered by UniqueIV with $p_1=10^{-07}$ and $p_2=0.01$ (beta, se, P-value: the GWAS estimates for each variant adjusted for age, sex, and 5 PCs, cGAUGE_p1 and cGAUGE_p2: thresholds used as input for the filter)

Supplementary Data 16: The instrument sets discovered by UniqueIV with $p_1=10^{-07}$ and $p_2=0.001$ (beta, se, P-value: the GWAS estimates for each variant adjusted for age, sex, and 5 PCs, cGAUGE_p1 and cGAUGE_p2: thresholds used as input for the filter).
